# Naringin Mitigates PEDV-Induced Intestinal Damage in Suckling Piglets by Modulating Inflammatory, Antiviral, and Metabolic and Transport Pathways

**DOI:** 10.3390/biom16010048

**Published:** 2025-12-28

**Authors:** Yanyan Zhang, Muzi Li, Zongyun Li, Zhonghua Li, Lei Wang, Di Zhao, Tao Wu, Dan Yi, Yongqing Hou

**Affiliations:** 1 Hubei Key Laboratory of Animal Nutrition and Feed Science, Wuhan Polytechnic University, Wuhan 430023, China; zhangyanyan@whpu.edu.cn (Y.Z.); 20230411054@whpu.edu.cn (M.L.); 20230411046@whpu.edu.cn (Z.L.); lzh1990@whpu.edu.cn (Z.L.); wanglei@whpu.edu.cn (L.W.); zhaodi@whpu.edu.cn (D.Z.); wutao@whpu.edu.cn (T.W.); yidan810204@whpu.edu.cn (D.Y.); 2 Engineering Research Center of Feed Protein Resources of Agricultural By-Products, Ministry of Education, Wuhan Polytechnic University, Wuhan 430023, China

**Keywords:** piglet, PEDV, intestine, naringin, crypt depth

## Abstract

This study evaluated the protective effects of naringin (NG) against intestinal injury in 7-day-old piglets infected with porcine epidemic diarrhea virus (PEDV). Eighteen piglets (Duroc × Landrace × Large, body weight = 2.58 ± 0.05 kg) were divided into three treatment groups based on similar body weights and equal numbers of males and females: the blank control group (CON group), the PEDV infection group (PEDV group), and the NG intervention + PEDV infection group (NG + PEDV group) (*n* = 6 per group). The experiment lasted for 11 days, comprising a pre-feeding period from days 0 to 3 and a formal experimental period from days 4 to 10. On days 4–10 of the experiment, piglets in the NG + PEDV group were orally administered NG (10 mg/kg). On Day 8 of the experiment, piglets in the PEDV and NG + PEDV groups were inoculated with PEDV (3 mL, 10^6^ 50% tissue culture infective dose (TCID_50_) per milliliter). On day 11 of the experiment, piglets were euthanized for sample collection. PEDV infection caused significant intestinal damage, including a decreased (*p* < 0.05) villus height in the duodenum and ileum and an increased (*p* < 0.05) crypt depth in all intestinal segments. This intestinal damage was accompanied by an impaired absorptive function, as indicated by reduced (*p* < 0.05) serum D-xylose. Further results showed that PEDV compromised the intestinal antioxidant capacity by decreasing (*p* < 0.05) glutathione peroxidase and catalase activities, and it stimulated the intestinal inflammatory response by upregulating (*p* < 0.05) the expression of key inflammatory genes, including regenerating family member 3 gamma (*REG3G*; duodenum, jejunum, colon), S100 calcium binding protein A9 (*S100A9*; ileum, colon), interleukin 1 beta (*IL-1β*; ileum, colon), and S100 calcium binding protein A8 (*S100A8*; colon). PEDV also suppressed the intestinal lipid metabolism pathway by downregulating (*p* < 0.05) the ileal expression of Solute Carrier Family 27 Member 4 (*SLC27A4*), Microsomal Triglyceride Transfer Protein (*MTTP*), Apolipoprotein A4 (*APOA4*), Apolipoprotein C3 (*APOC3*), Diacylglycerol O-Acyltransferase 1 (*DGAT1*), and Cytochrome P450 Family 2 Subfamily J Member 34 (*CYP2J34*). Moreover, PEDV suppressed the intestinal antiviral ability by downregulating (*p* < 0.05) interferon (*IFN*) signaling pathway genes, including MX dynamin like GTPase 1 (*MX1*) and *ISG15* ubiquitin like modifier (*ISG15*) in the duodenum; weakened intestinal water and ion transport by downregulating (*p* < 0.05) aquaporin 10 (*AQP10*) and potassium inwardly rectifying channel subfamily J member 13 (*KCNJ13*) in the duodenum, aquaporin 7 (*AQP7*) and transient receptor potential cation channel subfamily V member 6 (*TRPV6*) in the ileum, and *TRPV6* and transient receptor potential cation channel subfamily M member 6 (*TRPM6*) in the colon; and inhibited intestinal digestive and absorptive function by downregulating (*p* < 0.05) phosphoenolpyruvate carboxykinase 1 (*PCK1*) in the duodenum and sucrase-isomaltase (*SI*) in the ileum. Notably, NG effectively counteracted these detrimental effects. Moreover, NG activated the IFN signaling pathway in the jejunum and suppressed PEDV replication in the colon. In conclusion, NG alleviates PEDV-induced intestinal injury by enhancing the antioxidant capacity, suppressing inflammation, normalizing the expression of metabolic and transport genes, and improving the antiviral ability.

## 1. Introduction

The intestine is the primary site for nutrient digestion and absorption and constitutes the body’s largest immune organ. Consequently, a healthy intestinal environment is crucial for the optimal growth and development of young piglets [1]. Nonetheless, the underdeveloped intestinal function in neonatal piglets renders them highly susceptible to viral and bacterial infections that compromise the intestinal barrier. Enteroviruses, particularly porcine epidemic diarrhea virus (PEDV), are a predominant cause of intestinal damage under commercial production conditions. PEDV, a highly contagious coronavirus, induces porcine epidemic diarrhea (PED), a gastrointestinal disease characterized by severe diarrhea, vomiting, and dehydration [2]. The resultant poor growth performance, impaired digestive function, and high mortality rates, particularly in neonatal piglets, pose significant economic challenges to the swine industry [3,4]. Surviving piglets typically exhibit a stunted growth and reduced feed efficiency, further eroding producer profitability. Given the high genetic variability of PEDV, no universally effective vaccine or therapeutic agent is currently available for its prevention or control [5].

Plant-derived extracts, characterized by their complex mixtures of bioactive compounds, are renowned for their antioxidant and anti-inflammatory properties [6]. Historically employed in traditional medicine and food preservation, these extracts are increasingly being recognized as promising feed additives in animal nutrition, owing to their diverse biological activities [7]. Therefore, there is an urgent need to develop nutritional interventions that combine intestinal-protective properties with direct anti-PEDV activity. Citrus peel is rich in bioactive compounds, including natural antioxidants such as flavonoids [8]. Flavonoids exhibit multiple biological properties, including antioxidant, antibacterial, and anti-inflammatory effects [9]. Naringin (NG), the primary bitter component in citrus plants, is particularly abundant in grapefruit, bitter orange, and pomelo [10]. Research indicates that NG can significantly alter the thermal properties, rheological characteristics, and enzymatic behavior of potato starch. This opens up possibilities for developing starch-based foods with an improved texture and sustained-release carbohydrate functionality [11]. Previous studies have demonstrated that NG mitigates inflammation and oxidative stress by reducing pro-inflammatory factors, including interleukin-17 (*IL-17*) and nuclear factor kappa-B (*NF-κB*), and by enhancing the activity of antioxidant enzymes, such as superoxide dismutase [12,13]. Moreover, its anti-tumor mechanisms involve the promotion of apoptosis, inhibition of cancer cell migration, and modulation of the expression of pro-apoptotic genes, such as Bcl-2 and Caspase-3 [14]. NG has also been shown to improve cognitive and memory deficits by suppressing neuroinflammation, reducing oxidative stress, regulating metabolism, and inhibiting apoptosis [15]. Notably, NG inhibits the activation of the NLRP3 inflammasome, thereby alleviating lung inflammation during *Klebsiella pneumoniae* (Kpn) infection [16]. In models of colitis, NG reduces dextran sulfate sodium (DSS)-induced colonic injury and improves intestinal barrier dysfunction by modulating the expression of inflammation-related proteins [17]. Naringenin is the glycoside form of NG [18], and both compounds can interconvert within the body through enzymatic processes. Naringenin exhibits significant anti-PEDV activity [19]. Multiple computer simulation studies indicate that NG can effectively bind to several key proteins of Severe Acute Respiratory Syndrome Coronavirus 2 (SARS-CoV-2). This includes binding to the viral spike protein, with a binding affinity (−9.8 kcal/mol) that even surpasses that of the standard drug dexamethasone, potentially blocking viral attachment to host cell receptors [20]; and binding to the viral main protease (3CL-Mpro), directly impacting viral replication [21]. Research has further demonstrated that propolis extracts containing NG can inhibit SARS-CoV-2 infection of Vero E6 cells in vitro [22]. In summary, these properties render NG a multi-target therapeutic agent for treating various diseases.

Based on the commonalities within the *Coronaviridae* family shared by SARS-CoV-2 and PEDV (single-stranded positive-sense RNA, mucosal injury mechanisms) [21], combined with NG’s binding activity towards key SARS-CoV-2 proteins and the anti-PEDV properties of NG, it is hypothesized that “NG mitigates PEDV-induced intestinal injury in suckling piglets by exerting antiviral effects”. This provides a novel approach for developing broad-spectrum antiviral feed additives against coronaviruses. This study marks the first application of NG in a lactating piglet model infected with PEDV, evaluating its protective effects against PEDV-induced intestinal damage. The findings aim to provide a theoretical basis for incorporating NG into therapeutic regimens safeguarding intestinal health in PEDV-infected young pigs. Through this research, we endeavor to offer the piglet farming industry a low-cost, high-safety natural additive solution to mitigate the economic risks posed by PEDV.

## 2. Materials and Methods

### 2.1. Experimental Materials

PEDV (the Yunnan strain) was obtained from the Hubei Key Laboratory of Animal Nutrition and Feed Science, and the titer of the PEDV stock solution was adjusted to 10^6^ 50% tissue culture infective dose (TCID_50_) per milliliter [23]. The compound NG was procured from Shanghai Aladdin Biochemical Technology Co., Ltd. (Shanghai, China) (Catalog No.: N107345-100g). It had a purity of ≥95% and CAS No. of 10236-47-2. Experimental diet: modified whole milk powder (Nouriz, Shanghai, China).

### 2.2. Experimental Animals and Design

Eighteen healthy 7-day-old piglets (body weight = 2.58 ± 0.05 kg, equal numbers of males and females), derived from a crossbreed of Duroc × Landrace × Large White and sourced from PEDV-negative farms (Hubei Xufeng Agricultural Development Co., Ltd., Wuhan, China), were divided into three treatment groups based on similar body weights and equal numbers of males and females: the control group (CON group), the PEDV infection group (PEDV group), and the NG + PEDV infection group (NG + PEDV group) (*n* = 6 per group). The experiment lasted for 11 days, comprising a pre-feeding period from days 0 to 3 and a formal experimental period from days 4 to 10. On days 4–10 of the experiment, piglets in the NG + PEDV group received NG (10 mg/kg body weight dissolved in milk) [24,25,26,27,28,29,30] via oral administration at 19:30 each evening, while piglets in the CON and PEDV groups were administered an equal volume of milk. On day 8 of the experiment, piglets in the PEDV and NG + PEDV groups were orally administered 3 mL/head of PEDV virus solution (10^6^ TCID_50_/mL), while those in the CON group received an equal volume of Dulbecco’s Modified Eagle Medium (DMEM). From 22:00 on the 10th day until slaughter, all piglets were weaned off both feed and water. Piglets were weighed and recorded at 06:00 on days 0, 4, 8, and 11 to calculate the average daily gain (ADG). Following weighing at 06:00 on the 11th day, the piglets were administered D-xylose solution at a dose of 0.1 g/kg body weight via an oral drench. After 1 h, blood samples were collected. The administration of anesthetics in animal experimentation is a pivotal method for reconciling scientific rigor, operational practicality, and ethical considerations of animal welfare. Post-blood collection, Zoletil 50 (Zoletil 50, Virbac, Carros, France) was administered intramuscularly at a dose of 10 mg/kg body weight to the piglets. Slaughter and subsequent sample collection were conducted exclusively following the piglets’ complete loss of consciousness. Samples were immediately transferred to liquid nitrogen for rapid freezing and then stored at −80 °C for subsequent analysis.

### 2.3. Feeding Management

Piglets in each experimental group were housed in separate pens, with physical barriers between pens to prevent direct contact between groups, thereby minimizing the risk of cross-infection during the trial. A fresh air purification system was operational throughout the trial to ensure air circulation and cleanliness within the pens. The fresh air filtration level complied with laboratory animal husbandry standards, and the filtration units were cleaned regularly to maintain ventilation efficiency. Pen temperatures were strictly maintained at between 27 and 29 °C to ensure constant thermal conditions. The overall piggery environment was regulated at 30 °C ± 1 °C (optimal temperature 30 °C) to prevent temperature fluctuations from affecting piglet physiology. Relative humidity was consistently maintained at 60% through a coordinated use of humidifiers, dehumidifiers, and ventilation adjustments, providing an optimal growth environment. We implemented a timed, measured feeding regimen with five daily feedings at 07:30, 11:00, 15:00, 18:00, and 21:00. Piglets were administered an equal volume of reconstituted milk (prepared by mixing milk powder with water at a mass-to-volume ratio of 1:5) daily. Per pig per meal: 20 g milk powder (720 g reconstituted milk per group). Fresh drinking water was provided following the three main feeding periods (07:30, 15:00, 21:00), maintained at a suitable temperature to prevent cold water from irritating the intestines. The watering systems were constructed from sterile materials, cleaned and disinfected daily to ensure water hygiene and meet the physiological metabolic water requirements of piglets. Additionally, meticulous observations and records were made of the feeding behavior, daily feed intake, and mental state before and after each feeding session.

### 2.4. Preparation of Blood and Intestinal Samples

Blood samples were collected from the anterior vena cava of piglets using sterile needles and collection tubes. The whole blood was then processed for a complete blood cell count. The serum and plasma were separated by centrifugation at 3500 rpm for 15 min. These samples were cryopreserved at −80 °C for the analysis of biochemical parameters. Intestinal segments (approximately 2–3 cm in length) from the duodenum, jejunum, ileum, and colon were collected. Parts of the collected segments were immersed in paraformaldehyde fixative and underwent subsequent intestinal tissue section preparation. Other portions of collected intestinal tissues were thoroughly washed with physiological saline. Surface fat was removed under low-temperature conditions. Subsequently, the tissue was minced, wrapped in aluminum foil, labeled, and placed in gauze bags. It was then transferred to a liquid nitrogen tank for freezing and stored at −80 °C for future analysis.

### 2.5. Determination of Dao and D-Xylose Content

Diamine oxidase (DAO) and D-xylose detection kits (DAO, Catalog No. A088-1-1; D-xylose, Catalog No. A035-1-1) were procured from the Nanjing Jiancheng Bioengineering Institute (Nanjing, China). The testing procedures were conducted in accordance with the instructions provided.

### 2.6. Measurement of Intestinal Morphology

Intestinal tissue slices were prepared by Wuhan BOLF Biotechnology Co., Ltd. (Wuhan, China) Following Frankel’s methodology [31], five structurally intact villi were selected from each section for measurement. The intestinal morphology was evaluated using an Olympus BX-41 TF optical microscope (Tokyo, Japan) and the OLYMPUS cellSens standard 1.1.8 software (Olympus, Tokyo, Japan). The villus height (VH, vertical distance from the villus tip to the base), crypt depth (CD, vertical distance from the crypt opening to the villus base), and villus width (VW, lateral distance between the apical edges of epithelial cells on either side of the villus) were measured. Additionally, the VH/CD ratio was calculated.

### 2.7. Antioxidant Capacity Assay

The antioxidant capacity was measured using the following kits: catalase (CAT), Catalog No. A007-1-1; hydrogen peroxide (H_2_O_2_), Catalog No. A064-1-1; total superoxide dismutase (T-SOD), Catalog No. A001-1-2; malondialdehyde (MDA), Catalog No. A003-1-2; glutathione peroxidase (GSH-Px), Catalog No. A005-1-2; and myeloperoxidase (MPO), Catalog No. A044-1-1. All kits were procured from the Nanjing Jiancheng Bioengineering Institute (Nanjing, China).

### 2.8. Real-Time PCR

Total RNA was extracted using the RNAiso Plus Kit (Takara, Dalian, China) and then reverse-transcribed to cDNA using the PrimeScript™ RT Reagent Kit (Takara, Dalian, China) with gDNA Erase. The gene expression levels were measured using SYBR^®^ Premix Ex Taq™ (Tli RNaseH Plus) (Takara, Dalian, China) and the 7500 Fast Real-Time PCR System (Applied Biosystems, Foster City, California, USA). All reagents were procured from TaKaRa (Dalian, China). The ribosomal protein L19 (*RPL19*) gene was used as an internal control. The 2^−ΔΔCt^ method was used to calculate and statistically analyze the relative gene expression levels [32]. Primers used in this study are provided in Table 1.

### 2.9. Data Statistics and Analysis

Experimental data were compiled and analyzed using Microsoft Excel for Microsoft 365 software. One-way analysis of variance (ANOVA) and Turkey’s multiple range test were conducted using SPSS 26.0 statistical software. Daily feed intake data was calculated using the following formula: Group daily feed intake (g/day) = Group feed administered—Group feed leftovers—Group feed discarded. Data are presented as means ± standard error of the mean (SEM). Bar charts were generated using GraphPad Prism 9.5 software. A *p*-value of < 0.05 was deemed statistically significant.

### 2.10. AI Clarification

During the drafting of this manuscript, Chat GPT 4.0 was utilized for grammar checking, spelling checking, and language polishing.

## 3. Results

### 3.1. Effects of NG Administration on Growth Performance in PEDV-Infected Piglets

Data on daily feed intake is presented in Table 2. Data on average daily gain (ADG) is presented in Table 3. No significant difference in ADG was observed between the CON and PEDV groups from days 4 to 8. However, in the PEDV group, ADG was significantly decreased (*p* < 0.05). Compared with PEDV group, the NG + PEDV group exhibited an increasing trend in ADG.

### 3.2. Effect of NG Administration on Serum DAO and D-Xylose Levels in PEDV-Infected Piglets

To assess the effect of NG administration on intestinal integrity in PEDV-infected piglets, serum D-xylose levels and DAO activity were evaluated (Table 4). PEDV-infected piglets exhibited a significant impairment in intestinal function, characterized by a substantial decrease (*p* < 0.05) in serum D-xylose concentration. In contrast, administration of NG to PEDV-infected piglets showed a non-significant trend toward restoring normal D-xylose levels. No significant differences in serum DAO activity, a marker of intestinal barrier integrity, were detected among the three experimental groups.

### 3.3. Effects of NG Administration on Plasma Biochemical and Hematological Parameters in PEDV-Infected Piglets

Infection with PEDV led to significant alterations in plasma biochemical parameters compared to the CON group (Table 5). The PEDV group exhibited significantly elevated levels of total protein (TP) and blood urea nitrogen (BUN), accompanied by significantly decreased (*p* < 0.05) levels of total bilirubin (TB), alkaline phosphatase (ALP), and creatine kinase (CK) (*p* < 0.05). Administration of NG appeared to modulate these viral-induced changes. The NG + PEDV group demonstrated significantly increased (*p* < 0.05) concentrations of TB, albumin (ALB), triglycerides (TG), and low-density lipoprotein (LDL), with the concentration of CK remaining significantly lower (*p* < 0.05) than that of the CON group. Hematological analysis revealed that PEDV infection significantly increased (*p* < 0.05) both the absolute monocyte count (Mon) and the monocyte percentage (Mon (%)) compared to the CON group (Table 6).

### 3.4. Effect of NG Administration on Intestinal Morphology in PEDV-Infected Piglets

**Figure 1:**
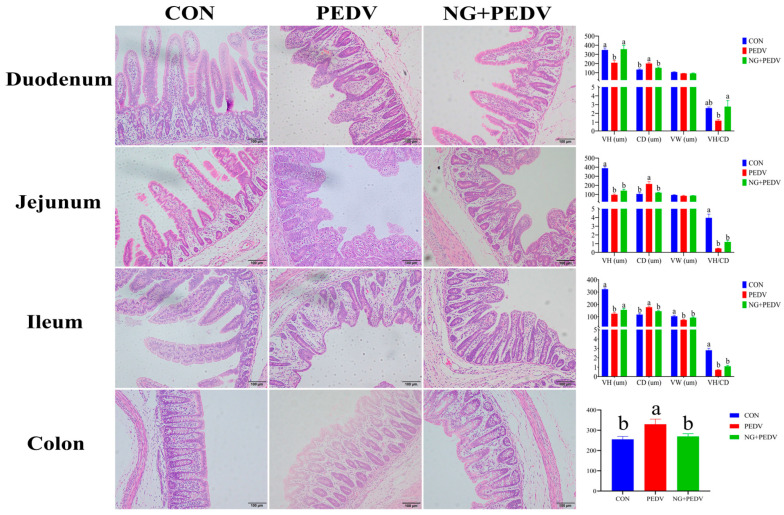
Effect of NG on the morphological structure of intestine in PEDV-infected piglets. VH: Villus height; CD: Crypt depth; VW: Villus width; VH/CD: Ratio of villus height to crypt depth. CON, the control group. PEDV, the PEDV infection group. NG + PEDV, the NG + PEDV infection group. Data are presented as means ± standard error of the mean for each group (*n* = 6). ^a, b^ Values within a column that do not share a common superscript letter indicate a significant difference at *p* < 0.05. Values with mixed superscript letters (^ab^) are not significantly different from the groups labeled with ^a^ or ^b^ alone.

Administration of NG to PEDV-infected piglets significantly altered the intestinal structure. Compared to the CON group, PEDV-infected piglets displayed marked increases (*p* < 0.05) in CD in the duodenum, jejunum, ileum, and colon. Conversely, VH and VH/VD were significantly reduced (*p* < 0.05) in the duodenum, jejunum, and ileum, as well as VW in the ileum. In contrast, piglets from the NG + PEDV group exhibited reduced (*p* < 0.05) CD in the duodenum, jejunum, ileum, and colon, along with significantly increased (*p* < 0.05) VH in the ileum and VH and VH/VD in the duodenum (Figure 1).

### 3.5. NG Administration Modulates Antioxidant Capacity in PEDV-Infected Piglets

**Figure 2:**
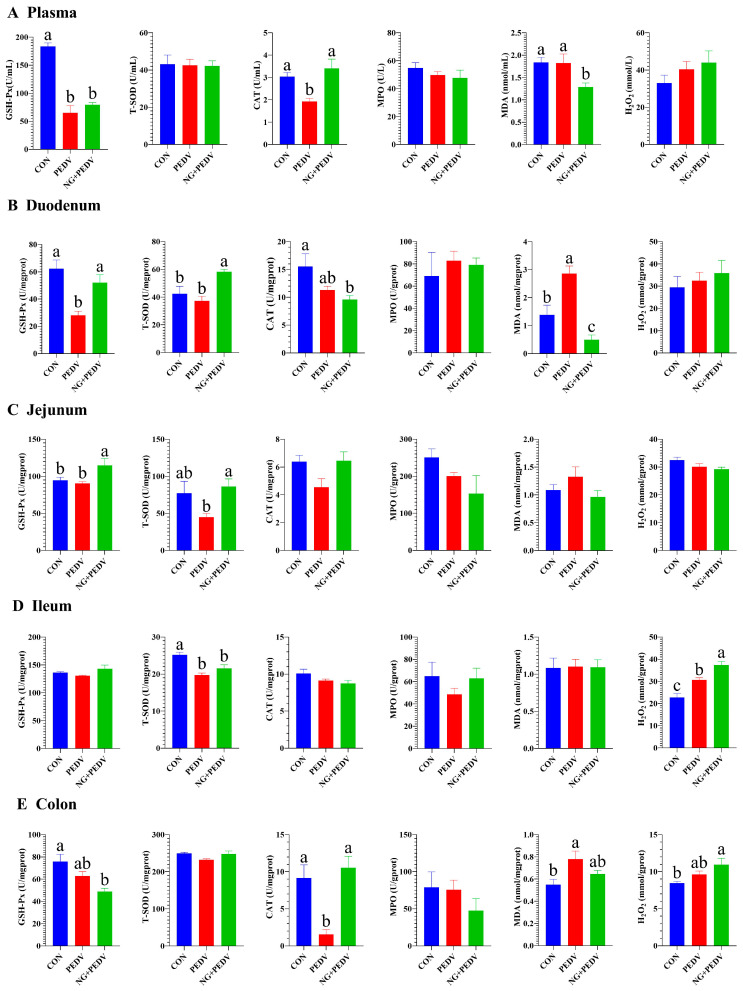
Effect of NG on the antioxidant capacity of PEDV-infected piglets. GSH-PX: Glutathione peroxidase; CAT: Catalase; T-SOD: Total superoxide dismutase; H_2_O_2_: Hydrogen peroxide; MDA: Malondialdehyde; MPO: Myeloperoxidase. CON, the control group. PEDV, the PEDV infection group. NG + PEDV, the NG + PEDV infection group. Data are presented as means ± standard error of the mean for each group (*n* = 6). ^a, b, c^ Values within a column that do not share a common superscript letter indicate a significant difference at *p* < 0.05. Values with mixed superscript letters (^ab^) are not significantly different from the groups labeled with ^a^ or ^b^ alone.

Antioxidant data is presented in Figure 2. PEDV-infected piglets exhibited significantly decreased (*p* < 0.05) plasma GSH-Px and CAT activities, duodenal GSH-Px activity, ileal T-SOD activity, and colonic CAT activity compared to the CON group. Furthermore, the levels of MDA in the duodenum and colon and H_2_O_2_ in the ileum were significantly higher (*p* < 0.05) in the PEDV group than in the CON group. In contrast, piglets in the NG + PEDV group demonstrated significantly increased (*p* < 0.05) plasma CAT activity, duodenal GSH-Px and T-SOD activities, jejunal GSH-Px and T-SOD activities, ileal H_2_O_2_ content, and colonic CAT activity. Additionally, plasma and duodenal MDA levels were significantly reduced (*p* < 0.05) in the NG + PEDV group.

### 3.6. Effects of NG Administration on Duodenal Functional-Gene Expression

**Figure 3:**
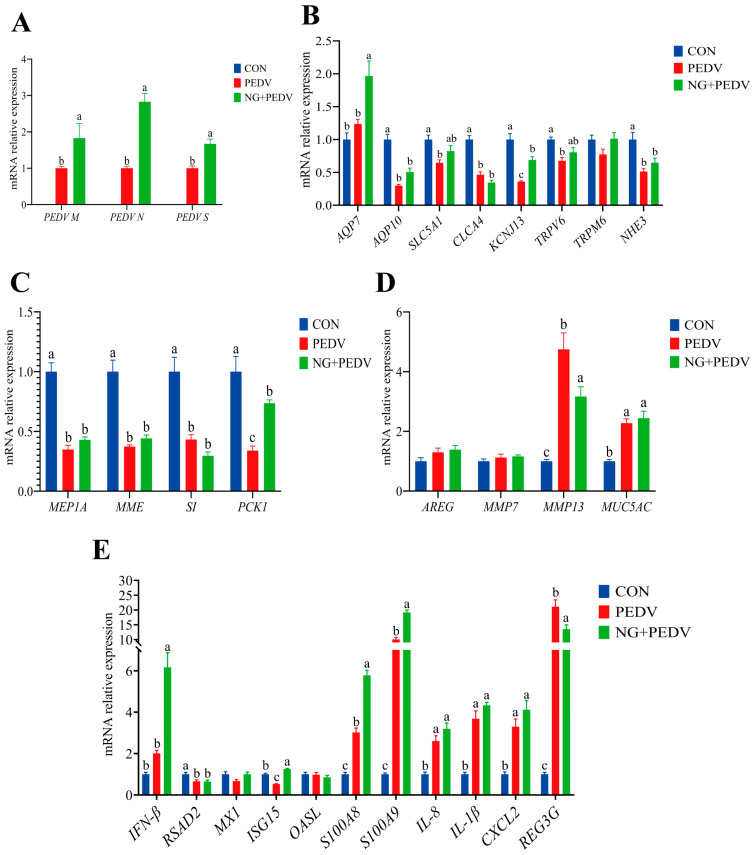
Effect of NG on duodenal-related genes in PEDV-infected piglets. (**A**) PEDV-associated genes; (**B**) Genes associated with water and ion transport channels; (**C**) Genes associated with digestion and absorption; (**D**) Genes associated with tissue injury and repair; (**E**) Genes associated with immunity and inflammation. *PEDV M*, Porcine Epidemic Diarrhea Virus Membrane Protein; *PEDV N*, Porcine Epidemic Diarrhea Virus Nucleocapsid Protein; *PEDV S*, Porcine Epidemic Diarrhea Virus Spike Protein; *AQP7*, Aquaporin 7; *AQP10*, Aquaporin 10; *SLC5A1*, Solute Carrier Family 5 Member 1; *CLCA4*, Chloride Channel Accessory 4; *KCNJ13*, Potassium Inwardly Rectifying Channel Subfamily J Member 13; *TRPM6*, Transient Receptor Potential Cation Channel Subfamily M Member 6; *TRPV6*, Transient Receptor Potential Cation Channel Subfamily V Member 6; *NHE3*, Sodium Hydrogen Exchanger 3; *MEP1A*, Meprin A Subunit Alpha; *MME*, Membrane Metalloendopeptidase; *SI*, Sucrase-Isomaltase; *PCK1*, Phosphoenolpyruvate Carboxykinase 1; *AREG*, Amphiregulin; *MMP7*, Matrix Metallopeptidase 7; *MMP13*, Matrix Metallopeptidase 13; *MUC5AC*, Mucin 5AC, Oligomeric Mucus/Gel-Forming; *IFN-β*, Interferon Beta; *RSAD2*, Radical S-Adenosyl Methionine Domain Containing 2; *MX1*, MX Dynamin Like GTPase 1; *ISG15*, Interferon Stimulated Gene 15; *OASL*, 2′,5′-Oligoadenylate Synthetase Like; *S100A8*, S100 Calcium Binding Protein A8; *S100A9*, S100 Calcium Binding Protein A9; *IL-8*, Interleukin 8; *IL-1β*, Interleukin 1 Beta; *CXCL2*, C-X-C Motif Chemokine Ligand 2; *REG3G*, Regenerating Family Member 3 Gamma. CON, the control group. PEDV, the PEDV infection group. NG + PEDV, the NG + PEDV infection group. Data are presented as mean ± standard error of the mean for each group (*n* = 6). ^a, b, c^ Values within a column that do not share a common superscript letter indicate a significant difference at *p* < 0.05. Values with mixed superscript letters (^ab^) are not significantly different from the groups labeled with ^a^ or ^b^ alone.

Data regarding duodenal gene expression levels are presented in Figure 3. In comparison to the PEDV group, mRNA expression levels of *PEDV M*, *PEDV N*, and *PEDV S* were significantly increased (*p* < 0.05) in the duodenum of the NG + PEDV group (Figure 3A). Compared to the CON group, mRNA expression levels of Aquaporin 10 (*AQP10*), Solute Carrier Family 5 Member 1 (*SLC5A1*), Chloride Channel Accessory 4 (*CLCA4*), Potassium Inwardly Rectifying Channel Subfamily J Member 13 (*KCNJ13*), Transient Receptor Potential Cation Channel Subfamily V Member 6 (*TRPV6*), and Sodium Hydrogen Exchanger 3 (*NHE3*) were significantly decreased (*p* < 0.05) in the duodenum of the PEDV group. In the NG + PEDV group, mRNA expression levels of Aquaporin 7 (*AQP7*) and *KCNJ13* were significantly elevated (*p* < 0.05) compared to those in the PEDV group (Figure 3B). Figure 3C illustrates that mRNA expression levels of Meprin A Subunit Alpha (*MEP1A*), Membrane Metalloendopeptidase (*MME*), Sucrase-Isomaltase (*SI*), and Phosphoenolpyruvate Carboxykinase 1 (*PCK1*) were significantly decreased (*p* < 0.05) in the duodenum of the PEDV group compared to the CON group. In the NG + PEDV group, the mRNA expression level of *PCK1* was significantly increased (*p* < 0.05) as compared to the PEDV group. Figure 3D shows that mRNA expression levels of Matrix Metallopeptidase 13 (*MMP13*) and Mucin 5AC (*MUC5AC*) were significantly increased (*p* < 0.05) in the duodenum of the PEDV group compared to the CON group. In contrast, the mRNA expression level of MMP13 was significantly decreased (*p* < 0.05) in the duodenum of the NG + PEDV group. In Figure 3E, mRNA expression levels of S100 Calcium Binding Protein A8 (*S100A8*), S100 Calcium Binding Protein A9 (*S100A9*), Interleukin 1 Beta (*IL-1β*), Interleukin 8 (*IL-8*), C-X-C Motif Chemokine Ligand 2 (*CXCL2*), and Regenerating Family Member 3 Gamma (*REG3G*) are significantly increased (*p* < 0.05) in the duodenum of the PEDV group compared to the CON group, whereas mRNA expression levels of Radical S-Adenosyl Methionine Domain Containing 2 (*RSAD2*) and Interferon Stimulated Gene 15 (*ISG15*) are significantly decreased (*p* < 0.05). In the NG + PEDV group, mRNA expression levels of Interferon Beta (*IFN-β*), *ISG15*, *S100A8*, and *S100A9* were significantly elevated (*p* < 0.05) in the duodenum, whereas mRNA expression of *REG3G* was significantly reduced (*p* < 0.05).

### 3.7. Effects of NG Administration on the Expression Levels on Jejunal Functional Genes

**Figure 4:**
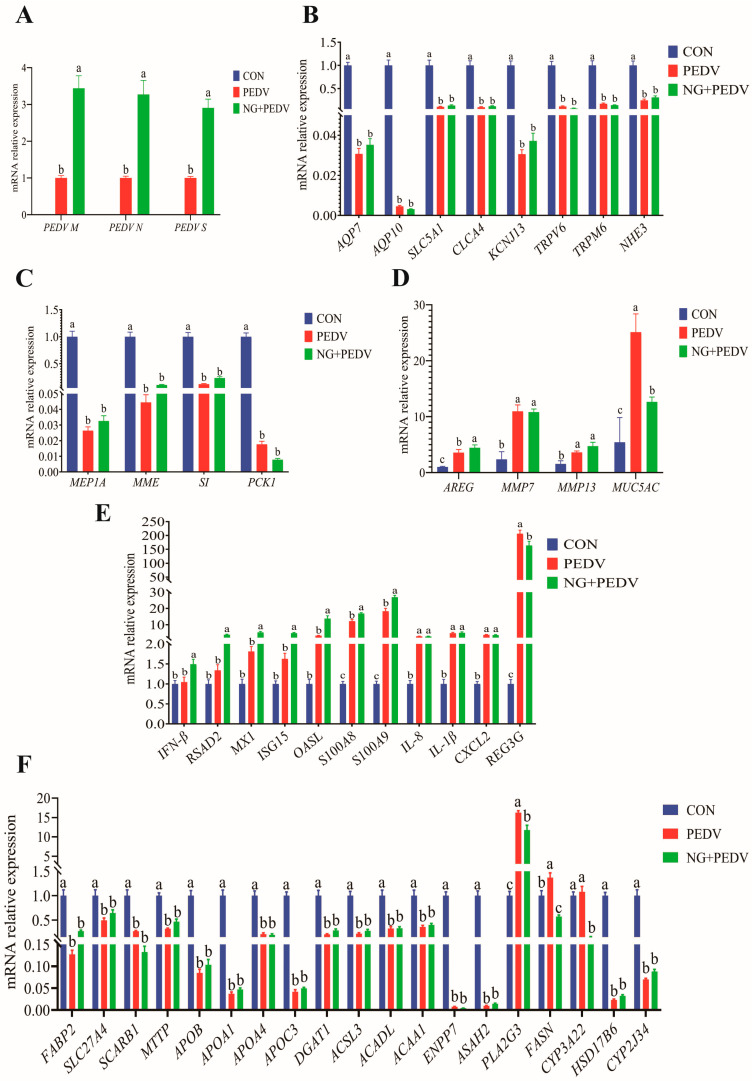
Effect of NG on jejunum-related genes in PEDV-infected piglets. (**A**) PEDV-related genes; (**B**) Genes associated with water and ion transport channels; (**C**) Genes related to digestion and absorption; (**D**) Genes linked to tissue injury and repair; (**E**) Genes involved in immunity and inflammation; (**F**) Genes associated with lipid metabolism. *PEDV M*, Porcine Epidemic Diarrhea Virus Membrane Protein; *PEDV N*, Porcine Epidemic Diarrhea Virus Nucleocapsid Protein; *PEDV S*, Porcine Epidemic Diarrhea Virus Spike Protein; *AQP7*, Aquaporin 7; *AQP10*, Aquaporin 10; *SLC5A1*, Solute Carrier Family 5 Member 1; *CLCA4*, Chloride Channel Accessory 4; *KCNJ13*, Potassium Inwardly Rectifying Channel Subfamily J Member 13; *TRPM6*, Transient Receptor Potential Cation Channel Subfamily M Member 6; *TRPV6*, Transient Receptor Potential Cation Channel Subfamily V Member 6; *NHE3*, Sodium Hydrogen Exchanger 3; *MEP1A*, Meprin A Subunit Alpha; *MME*, Membrane Metalloendopeptidase; *SI*, Sucrase-Isomaltase; *PCK1*, Phosphoenolpyruvate Carboxykinase 1; *AREG*, Amphiregulin; *MMP7*, Matrix Metallopeptidase 7; *MMP13*, Matrix Metallopeptidase 13; *MUC5AC*, Mucin 5AC, Oligomeric Mucus/Gel-Forming; *IFN-β*, Interferon Beta; *RSAD2*, Radical S-Adenosyl Methionine Domain Containing 2; *MX1*, MX Dynamin Like GTPase 1; *ISG15*, Interferon Stimulated Gene 15; *OASL*, 2′,5′-Oligoadenylate Synthetase Like; *S100A8*, S100 Calcium Binding Protein A8; *S100A9*, S100 Calcium Binding Protein A9; *IL-8*, Interleukin 8; *IL-1β*, Interleukin 1 Beta; *CXCL2*, C-X-C Motif Chemokine Ligand 2; *REG3G*, Regenerating Family Member 3 Gamma; *FABP2*, Fatty Acid Binding Protein 2, Intestinal; *SLC27A4*, Solute Carrier Family 27 Member 4; *SCARB1*, Scavenger Receptor Class B Member 1; *MTTP*, Microsomal Triglyceride Transfer Protein; *APOB*, Apolipoprotein B; *APOA1*, Apolipoprotein A1; *APOA4*, Apolipoprotein A4; *APOC3*, Apolipoprotein C3; *DGAT1*, Diacylglycerol O-Acyltransferase 1; *ACSL3*, Acyl-CoA Synthetase Long Chain Family Member 3; *ACADL*, Acyl-CoA Dehydrogenase, Long Chain; *ACAA1*, Acetyl-CoA Acyltransferase 1; *ENPP7*, Ectonucleotide Pyrophosphatase/Phosphodiesterase Family Member 7; *ASAH2*, N-Acylsphingosine Amidohydrolase 2; *PLA2G3*, Phospholipase A2 Group III; *FASN*, Fatty Acid Synthase; *CYP3A22*, Cytochrome P450 Family 3 Subfamily A Member 22; *HSD17B6*, Hydroxysteroid 17-Beta Dehydrogenase 6; *CYP2J34*, Cytochrome P450 Family 2 Subfamily J Member 34. CON, the control group. PEDV, the PEDV infection group. NG + PEDV, the NG + PEDV infection group. Data are presented as mean ± standard error of the mean for each group (*n* = 6). ^a, b, c^ Values within a column that do not share a common superscript letter indicate a significant difference at *p* < 0.05.

Gene expression levels in the jejunum are summarized in Figure 4. Figure 4A demonstrates that mRNA expression of *PEDV M*, *PEDV N*, and *PEDV S* was significantly higher (*p* < 0.05) in the jejunum of the NG + PEDV group compared to the PEDV group. In contrast, mRNA expression levels of *AQP7*, *AQP10*, *SLC5A1*, *CLCA4*, *KCNJ13*, *TRPV6*, Transient Receptor Potential Cation Channel Subfamily M Member 6 (*TRPM6*), and *NHE3* were significantly lower (*p* < 0.05) in the jejunum of PEDV group animals compared to the CON group (Figure 4B). Figure 4C reveals that mRNA expression of *MEP1A*, *MME*, *SI*, and *PCK1* was significantly diminished (*p* < 0.05) in the jejunum of PEDV group animals relative to the CON group. Figure 4D indicates that mRNA expression of Amphiregulin (*AREG*), Matrix Metallopeptidase 7 (*MMP7*), *MMP13*, and *MUC5AC* was significantly elevated (*p* < 0.05) in the jejunum of PEDV group animals, compared to the CON group. In the NG + PEDV group, mRNA expression levels of *AREG* were significantly increased (*p* < 0.05), whereas *MUC5AC* mRNA expression was significantly decreased (*p* < 0.05) compared to the PEDV group. Figure 4E shows that mRNA expression levels of *S100A8*, *S100A9*, *IL-1β*, *IL-8*, *CXCL2*, and *REG3G* were significantly increased (*p* < 0.05) in the jejunum of PEDV group piglets when compared to the CON group. In the NG + PEDV group, mRNA expression levels of *IFN-β*, *RSAD2*, *MX1*, *ISG15*, 2′,5′-Oligoadenylate Synthetase Like (*OASL*), *S100A8*, and *S100A9* were significantly elevated (*p* < 0.05), whereas *REG3G* mRNA expression was significantly reduced (*p* < 0.05) compared to the PEDV group. Figure 4F illustrates that mRNA expression levels of Fatty Acid Binding Protein 2, Intestinal (*FABP2*), Solute Carrier Family 27 Member 4 (*SLC27A4*), Scavenger Receptor Class B Member 1 (*SCARB1*), Microsomal Triglyceride Transfer Protein (*MTTP*), Apolipoprotein B (*APOB*), Apolipoprotein A1 (*APOA1*), Apolipoprotein A4 (*APOA4*), Apolipoprotein C3 (*APOC3*), Diacylglycerol O-Acyltransferase 1 (*DGAT1*), Acyl-CoA Synthetase Long Chain Family Member 3 (*ACSL3*), Acyl-CoA Dehydrogenase, Long Chain (*ACADL*), Acetyl-CoA Acyltransferase 1 (*ACAA1*), Ectonucleotide Pyrophosphatase/Phosphodiesterase Family Member 7 (*ENPP7*), N-Acylsphingosine Amidohydrolase 2 (*ASAH2*), Hydroxysteroid 17-Beta Dehydrogenase 6 (*HSD17B6*), and Cytochrome P450 Family 2 Subfamily J Member 34 (*CYP2J34*) were significantly reduced (*p* < 0.05), while Phospholipase A2 Group III (*PLA2G3*) and Fatty Acid Synthase (*FASN*) mRNA expression levels were significantly increased (*p* < 0.05) in the jejunum of PEDV group animals compared to the CON group. Conversely, mRNA expression levels of *PLA2G3*, *FASN*, and Cytochrome P450 Family 3 Subfamily A Member 22 (*CYP3A22*) were significantly reduced (*p* < 0.05) in the jejunum of NG + PEDV group animals compared to the PEDV group.

### 3.8. Effects of NG Administration on the Expression Levels on Ileal Functional Genes

**Figure 5:**
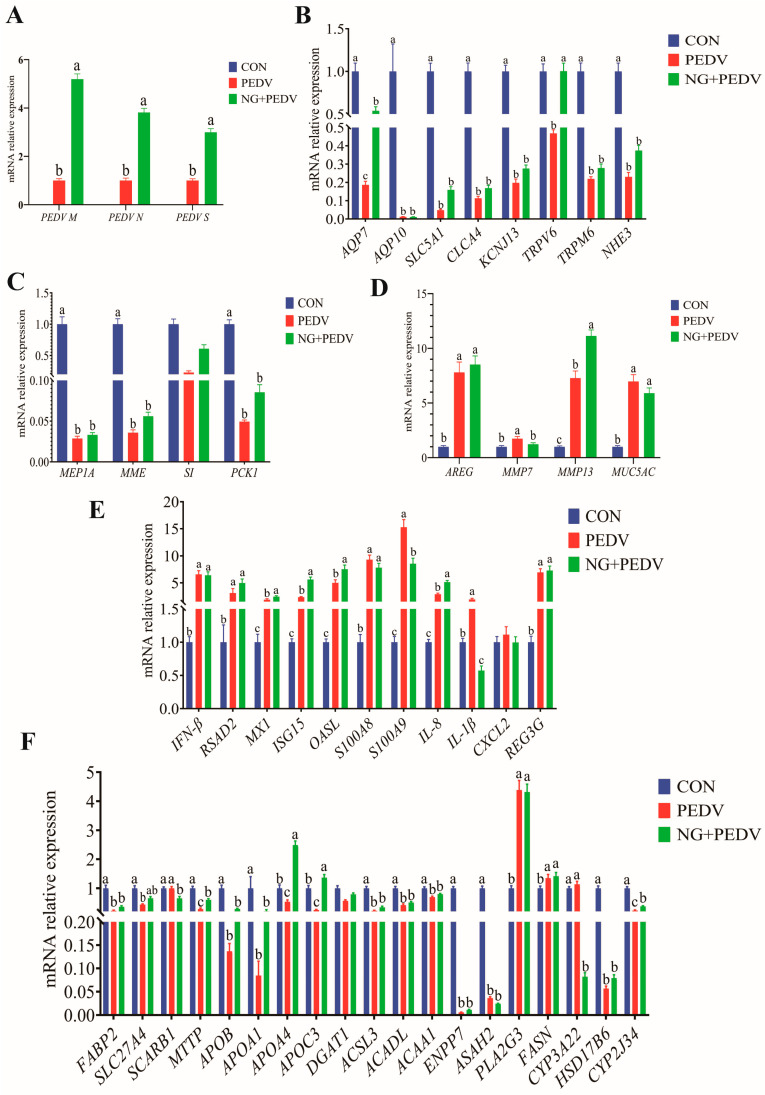
Effect of NG on ileus-related genes in PEDV-infected piglets. (**A**) PEDV-related genes; (**B**) Genes associated with water and ion transport channels; (**C**) Genes related to digestion and absorption; (**D**) Genes linked to tissue injury and repair; (**E**) Genes involved in immunity and inflammation; (**F**) Genes associated with lipid metabolism. *PEDV M*, Porcine Epidemic Diarrhea Virus Membrane Protein; *PEDV N*, Porcine Epidemic Diarrhea Virus Nucleocapsid Protein; *PEDV S*, Porcine Epidemic Diarrhea Virus Spike Protein; *AQP7*, Aquaporin 7; *AQP10*, Aquaporin 10; *SLC5A1*, Solute Carrier Family 5 Member 1; *CLCA4*, Chloride Channel Accessory 4; *KCNJ13*, Potassium Inwardly Rectifying Channel Subfamily J Member 13; *TRPM6*, Transient Receptor Potential Cation Channel Subfamily M Member 6; *TRPV6*, Transient Receptor Potential Cation Channel Subfamily V Member 6; *NHE3*, Sodium Hydrogen Exchanger 3; *MEP1A*, Meprin A Subunit Alpha; *MME*, Membrane Metalloendopeptidase; *SI*, Sucrase-Isomaltase; *PCK1*, Phosphoenolpyruvate Carboxykinase 1; *AREG*, Amphiregulin; *MMP7*, Matrix Metallopeptidase 7; *MMP13*, Matrix Metallopeptidase 13; *MUC5AC*, Mucin 5AC, Oligomeric Mucus/Gel-Forming; *IFN-β*, Interferon Beta; *RSAD2*, Radical S-Adenosyl Methionine Domain Containing 2; *MX1*, MX Dynamin Like GTPase 1; *ISG15*, Interferon Stimulated Gene 15; *OASL*, 2′,5′-Oligoadenylate Synthetase Like; *S100A8*, S100 Calcium Binding Protein A8; *S100A9*, S100 Calcium Binding Protein A9; *IL-8*, Interleukin 8; *IL-1β*, Interleukin 1 Beta; *CXCL2*, C-X-C Motif Chemokine Ligand 2; *REG3G*, Regenerating Family Member 3 Gamma; *FABP2*, Fatty Acid Binding Protein 2, Intestinal; *SLC27A4*, Solute Carrier Family 27 Member 4; *SCARB1*, Scavenger Receptor Class B Member 1; *MTTP*, Microsomal Triglyceride Transfer Protein; *APOB*, Apolipoprotein B; *APOA1*, Apolipoprotein A1; *APOA4*, Apolipoprotein A4; *APOC3*, Apolipoprotein C3; *DGAT1*, Diacylglycerol O-Acyltransferase 1; *ACSL3*, Acyl-CoA Synthetase Long Chain Family Member 3; *ACADL*, Acyl-CoA Dehydrogenase, Long Chain; *ACAA1*, Acetyl-CoA Acyltransferase 1; *ENPP7*, Ectonucleotide Pyrophosphatase/Phosphodiesterase Family Member 7; *ASAH2*, N-Acylsphingosine Amidohydrolase 2; *PLA2G3*, Phospholipase A2 Group III; *FASN*, Fatty Acid Synthase; *CYP3A22*, Cytochrome P450 Family 3 Subfamily A Member 22; *HSD17B6*, Hydroxysteroid 17-Beta Dehydrogenase 6; *CYP2J34*, Cytochrome P450 Family 2 Subfamily J Member 34. CON, the control group. PEDV, the PEDV infection group. NG + PEDV, the NG + PEDV infection group. Data are presented as mean ± standard error of the mean for each group (*n* = 6). ^a^, ^b^, ^c^ Values within a column that do not share a common superscript letter indicate significant differences (*p* < 0.05).

Gene expression data pertinent to the ileum is presented in Figure 5. Figure 5A indicates that mRNA expression levels of *PEDV M*, *PEDV N*, and *PEDV S* were significantly increased (*p* < 0.05) in the ileum of piglets from the NG + PEDV group compared to the PEDV group. In contrast, the ileum of piglets in the PEDV group exhibited significantly reduced (*p* < 0.05) mRNA expression levels of *AQP7*, *AQP10*, *SLC5A1*, *CLCA4*, *KCNJ13*, *TRPV6*, *TRPM6*, and *NHE3* relative to the CON group. In the NG + PEDV group, mRNA expression levels of *AQP7* and *TRPV6* were significantly elevated (*p* < 0.05) in the ileum when compared to the PEDV group (Figure 5B). Figure 5C reveals that in the PEDV group, mRNA expression levels of *MEP1A*, *MME*, *SI*, and *PCK1* were significantly decreased (*p* < 0.05) in the ileum when compared to the CON group. In the NG + PEDV group, mRNA expression levels of *SI* were significantly increased (*p* < 0.05) in the ileum relative to the PEDV group. Figure 5D demonstrates that mRNA expression levels of *AREG*, *MMP7*, *MMP13*, and *MUC5AC* were significantly increased (*p* < 0.05) in the ileum of PEDV group piglets compared to the CON group. In the NG + PEDV group, mRNA expression levels of *MMP7* were significantly decreased (*p* < 0.05), whereas those of *MMP13* were significantly increased (*p* < 0.05) in the ileum when compared to the PEDV group. Figure 5E illustrates that in the PEDV group, mRNA expression levels of *IFN-β*, *MX1*, *ISG15*, *OASL*, *S100A8*, *S100A9*, *IL-1β*, *IL-8*, and *REG3G* were significantly elevated (*p* < 0.05) in the ileum of piglets relative to the CON group. In contrast, the NG + PEDV group exhibited significantly increased (*p* < 0.05) mRNA expression levels of *MX1*, *ISG15*, *OASL*, and *IL-8*, with significantly reduced (*p* < 0.05) levels of *S100A9* and *IL-1β* in the ileum when compared to the PEDV group. Figure 5F shows that in the PEDV group, mRNA expression levels of *FABP2*, *SLC27A4*, *MTTP*, *APOB*, *APOA1*, *APOA4*, *APOC3*, *DGAT1*, *ACSL3*, *ACADL*, *ACAA1*, *ENPP7*, *ASAH2*, *HSD17B6*, and *CYP2J34* were significantly reduced (*p* < 0.05), while *PLA2G3* and *FASN* were significantly increased (*p* < 0.05) in the ileum of piglets. In the NG + PEDV group, mRNA expression levels of *MTTP*, *APOA4*, *APOC3*, *DGAT1*, and *CYP2J34* were significantly increased (*p* < 0.05), whereas those of *SCARB1* and *CYP3A22* significantly decreased (*p* < 0.05) in the ileum when compared to the PEDV group.

### 3.9. Effects of NG Administration on Colonic Functional Gene Expression

**Figure 6:**
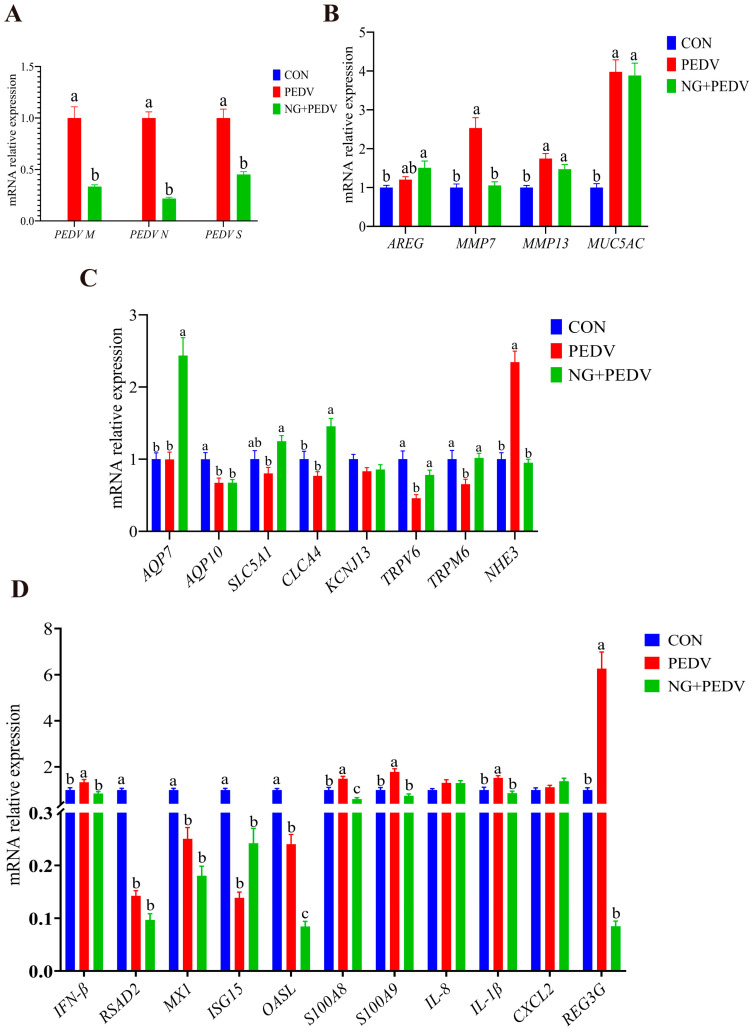
Effects of NG on colon-related gene expression in PEDV-infected piglets. (**A**) PEDV-related genes; (**B**) Genes associated with tissue injury and repair; (**C**) Genes related to water and ion transport channels; (**D**) Genes linked to immunity and inflammation. *PEDV M*, Porcine Epidemic Diarrhea Virus Membrane Protein; *PEDV N*, Porcine Epidemic Diarrhea Virus Nucleocapsid Protein; *PEDV S*, Porcine Epidemic Diarrhea Virus Spike Protein; *AREG*, Amphiregulin; *MMP7*, Matrix Metallopeptidase 7; *MMP13*, Matrix Metallopeptidase 13; *MUC5AC*, Mucin 5AC, Oligomeric Mucus/Gel-Forming; *AQP7*, Aquaporin 7; *AQP10*, Aquaporin 10; *SLC5A1*, Solute Carrier Family 5 Member 1; *CLCA4*, Chloride Channel Accessory 4; *KCNJ13*, Potassium Inwardly Rectifying Channel Subfamily J Member 13; *TRPM6*, Transient Receptor Potential Cation Channel Subfamily M Member 6; *TRPV6*, Transient Receptor Potential Cation Channel Subfamily V Member 6; *NHE3*, Sodium Hydrogen Exchanger 3; *IFN-β*, Interferon Beta; *RSAD2*, Radical S-Adenosyl Methionine Domain Containing 2; *MX1*, MX Dynamin Like GTPase 1; *ISG15*, Interferon Stimulated Gene 15; *OASL*, 2′,5′-Oligoadenylate Synthetase Like; *S100A8*, S100 Calcium Binding Protein A8; *S100A9*, S100 Calcium Binding Protein A9; *IL-8*, Interleukin 8; *IL-1β*, Interleukin 1 Beta; *CXCL2*, C-X-C Motif Chemokine Ligand 2; *REG3G*, Regenerating Family Member 3 Gamma. CON, the control group. PEDV, the PEDV infection group. NG + PEDV, the NG + PEDV infection group. Data is presented as mean ± standard error of the mean (SEM) for each group (*n* = 6). Significant differences among groups are indicated by different superscript letters ^a^, ^b^, ^c^ within a column (*p* < 0.05).

Gene expression levels associated with the colon are summarized in Figure 6. Figure 6A illustratea that mRNA expression levels of *PEDV M*, *PEDV N*, and *PEDV S* were significantly diminished (*p* < 0.05) in the colons of piglets from the NG + PEDV group when compared to the PEDV group. Conversely, in the PEDV group, mRNA expression levels of *MMP7*, *MMP13*, and *MUC5AC* were substantially elevated (*p* < 0.05) compared to the CON group. In the NG + PEDV group, mRNA expression levels of *MMP7* were significantly decreased (*p* < 0.05) relative to the PEDV group (Figure 6B). Figure 6C reveals that in the PEDV group, mRNA expression levels of *AQP10*, *TRPV6*, and *TRPM6* were notably reduced (*p* < 0.05), whereas *NHE3* expression was significantly increased (*p* < 0.05) compared to the CON group. In contrast, the NG + PEDV group exhibited significant increases (*p* < 0.05) in mRNA expression levels of *AQP7*, *SLC5A1*, *CLCA4*, *TRPV6*, and *TRPM6*, along with a decrease in *NHE3* expression, when compared to the PEDV group. Figure 6D indicates that in the PEDV group, mRNA expression levels of *IFN-β*, *S100A8*, *S100A9*, *IL-1β*, and *REG3G* were significantly increased (*p* < 0.05), whereas *RSAD2*, *MX1*, *ISG15*, and *OASL* were significantly decreased (*p* < 0.05) when compared to the CON group. In the NG + PEDV group, mRNA expression levels of *IFN-β*, *OASL*, *S100A8*, *S100A9*, *IL-1β*, and *REG3G* were significantly reduced (*p* < 0.05) relative to the PEDV group.

## 4. Discussion

The D-xylose absorption test is a standard diagnostic tool for evaluating intestinal mucosal integrity and absorptive capacity [33]. In line with previous research [34], PEDV infection in piglets resulted in a substantial decrease in serum D-xylose concentrations, thereby confirming the virus’s ability to compromise the intestinal barrier and impair absorptive function. Nonetheless, the administration of NG led to an increase in serum D-xylose levels, providing direct evidence of a restored intestinal absorption function. This analysis was expanded to include key enzymes involved in digestion and carbohydrate metabolism. *SI*, an enzyme situated at the small intestinal brush border, is essential for the hydrolysis of sucrose and maltose into absorbable monosaccharides [35,36]. Moreover, *PCK1* is a rate-limiting enzyme in gluconeogenesis, pivotal for maintaining glucose homeostasis [37]. The findings of this study indicate that PEDV infection significantly downregulated the relative mRNA expression of both *SI* and *PCK1*, which may have exacerbated carbohydrate malabsorption and metabolic dysfunction. NG administration reversed these effects, significantly increasing *SI* expression in the ileum and *PCK1* expression in the duodenum. These results suggest that NG mitigates PEDV-induced absorptive dysfunction, at least in part, by modulating the expression of key enzymes involved in intestinal digestion and glucose metabolism. Furthermore, this study investigated the transmembrane transport of water and ions, a critical component of intestinal function. Notably, PEDV infection significantly reduced the intestinal mRNA expression of key transport proteins, including the aquaporins *AQP7* and *AQP10*, which are essential for water transport [38]. Similarly, the expression of several ion channels was downregulated: *TRPV6* [39], *TRPM6* [40], and *KCNJ13* [41]. The observed downregulation of *TRPV6* and *KCNJ13* is consistent with previous reports on PEDV-infected piglets 39. This extensive suppression of genes critical for water homeostasis and the electrolyte balance further underscores the capacity of PEDV to induce intestinal damage in piglets. Following NG administration, there was a significant upregulation in the intestinal expression of key ion channel and aquaporin genes. Specifically, transcript levels of *AQP10* and *KCNJ13* were elevated in the duodenum, *AQP7* in the ileum, and *TRPV6* and *TRPM6* in the colon. These findings demonstrate the ability of NG to mitigate PEDV-induced intestinal damage in piglets.

PEDV infection elicited a systemic immune-inflammatory response in piglets, as evidenced by significantly increased Mon levels. Simultaneously, PEDV infection initiated a robust intestinal inflammatory response, characterized by the upregulation of several critical mediators, including the pro-inflammatory cytokines *S100A8* and *S100A9* [42], *IL-1β* [43], and *REG3G* [44,45]. NG administration effectively mitigated this inflammation in a region-specific fashion. In the duodenum, *S100A8* and *REG3G* expression was downregulated, whereas in the ileum, *S100A9* and *IL-1β* levels were reduced. The colon exhibited the most substantial impact, with NG markedly suppressing all inflammatory markers induced by PEDV infection. Moreover, NG’s influence on PEDV replication was also region-dependent. Viral replication was enhanced in the duodenum, jejunum, and ileum but was significantly suppressed in the colon, aligning with the strong anti-inflammatory effects of NG observed in this region.

Intestinal health and function are typically evaluated through key morphometric indices, such as VH, VW, CD, and VH/CD. In the present study, infection with PEDV led to severe disruption of the intestinal barrier, as evidenced by significant reductions in VH and VW, a significant increase in CD, and a consequent decrease in the VH/CD ratio. These findings underscore the profound structural damage to the intestinal mucosa induced by PEDV. Post-administration of NG, piglets exhibited a partial reversal of the intestinal damage. NG treatment resulted in a significant increase in duodenal VH and a notable reduction in CD, suggesting that the compound effectively promotes the repair of PEDV-induced morphological injuries. To elucidate the molecular mechanisms underlying these structural changes, this study quantified the intestinal mRNA expression of *MMP7* and *MMP13*. These zinc-dependent endopeptidases are crucial in extracellular matrix degradation and tissue remodeling and are implicated in the tissue destruction observed in inflammatory conditions such as rheumatoid arthritis [46]. The results demonstrated that PEDV infection significantly upregulated the relative mRNA expression of both *MMP7* and *MMP13* in the intestinal tissues of infected piglets. In contrast, NG administration significantly downregulated *MMP7* expression in the ileum and colon, along with *MMP13* expression in the duodenum. These findings suggest that NG mitigates intestinal tissue damage and facilitates morphological repair by inhibiting the expression of these pivotal metalloproteinases.

GSH-Px, T-SOD, and CAT are pivotal enzymes in the antioxidant defense system of piglets. They protect cells from oxidative damage by scavenging reactive oxygen species (ROS) and free radicals. MDA, a byproduct of lipid peroxidation, is a critical biomarker for oxidative damage [47]. PEDV infection significantly diminishes the activities of GSH-Px, T-SOD, and CAT in piglet plasma and intestines, while it markedly increases MDA levels, indicative of severe oxidative stress. This finding aligns with previous research [48]. Nonetheless, following oral administration of NG, the activities of GSH-Px and CAT were significantly enhanced, whereas MDA levels were notably reduced. These outcomes suggest that NG effectively mitigates PEDV-induced oxidative stress by augmenting the host’s antioxidant capacity and diminishing the accumulation of oxidative byproducts.

Normal intestinal lipid metabolism is regulated by a constellation of pivotal genes. Notably, these include *SLC27A4*, a fatty acid transporter pivotal for the uptake and metabolism of long-chain fatty acids; *MTTP*, which encodes a protein essential for lipoprotein particle formation and lipid transport; *APOA4*, an apolipoprotein primarily synthesized in the intestine to facilitate fat absorption and regulate triglyceride levels; *APOC3*, an apolipoprotein that inhibits lipoprotein lipase activity and modulates triglyceride catabolism; *DGAT1*, encoding an endoplasmic reticulum enzyme that catalyzes triglyceride synthesis; and *CYP2J34*, a cytochrome P450 enzyme implicated in endogenous lipid and hormone metabolism [49,50,51,52,53]. PEDV infection resulted in a significant downregulation of the mRNA expression of *SLC27A4*, *MTTP*, *APOA4*, *APOC3*, and *DGAT1* in the jejunum. In the ileum, the expression of all six genes, including *CYP2J34*, was also significantly diminished. NG administration mitigated these alterations, indicating that NG may alleviate PEDV-induced intestinal lipid metabolism disorders. This suggests that NG could potentially mitigate PEDV-induced intestinal lipid metabolism disorders by restoring the expression of these crucial genes, thereby providing energy for intestinal repair.

The experimental results, in conjunction with the existing literature, indicate that the repair mechanism of NG in ameliorating PEDV-induced intestinal damage in piglets involved a complex interplay of interconnected pathways. Initially, NG may modulate the S100A8 signaling pathway. Prior research utilizing a dextran sulfate sodium (DSS)-induced colitis model revealed that increased *S100A8* expression is concurrent with intestinal damage, while the inhibition of the S100A8-TLR4-NF-κB axis effectively reduces intestinal damage and restores a normal architecture [54]. In this study, NG was observed to downregulate *S100A8* expression, suggesting it may exert potential protective effects through anti-inflammatory mechanisms. Secondly, NG appeared to regulate *MMP7* and Paneth cell function. *MMP7*, secreted by Paneth cells in the intestine, has a positive correlation with Paneth cell abundance and CD [55]. Given that PEDV infection upregulated *MMP7*, potentially leading to increased tissue degradation, the downregulation of *MMP7* by NG may improve the crypt morphology by modulating Paneth cell activity. Additionally, NG enhanced the antioxidant capacity of the intestine. GSH-Px, an antioxidant enzyme active in the crypt region, and the exogenous administration of CAT have been shown to reduce CD and improve the VH/CD in weaned piglets [56,57]. Our findings demonstrated that NG increased the activity of both GSH-Px and CAT, thereby strengthening antioxidant defenses within the intestinal crypts. In conclusion, these findings suggest that NG mitigates the adverse effects of PEDV on the intestine by synergistically downregulating *S100A8* and *MMP7* while upregulating GSH-Px and CAT activity, ultimately leading to intestinal repair.

This research demonstrates that NG reduces the expression of intestinal inflammatory genes, with this effect being particularly pronounced in the colon. Concurrently, NG also inhibits the replication of PEDV within the colon. Morphologically, NG improved the intestinal architecture by reducing CD. Additionally, NG strengthens intestinal antioxidant defenses by enhancing the activity of GSH-Px and CAT and lowering the levels of the oxidative stress marker MDA. Metabolically, NG corrected dysregulation by upregulating the expression of various lipid metabolism-related genes in the jejunum and ileum, thereby supporting the energy requirements for tissue repair. Collectively, these synergistic effects contribute to the comprehensive repair of the damaged intestine in PEDV-infected piglets. Although the experimental results of this study indicate that NG can significantly improve the intestinal health of PEDV-infected piglets, larger-scale and longer-term trials are required to validate the safety and efficacy of NG. Furthermore, mechanism studies to clarify its target sites are necessary to provide a more robust basis for its practical application.

## 5. Conclusions

This research confirms that NG effectively mitigates PEDV-induced intestinal damage in piglets, supporting its potential as a therapeutic for PEDV-associated enteropathy. PEDV infection impaired the intestinal function (reduced serum D-xylose, downregulated key metabolic/transport genes), as well as triggered inflammation, oxidative stress, and structural damage. NG reversed these deficits: restored intestinal function and gene expression, exerted region-specific anti-inflammatory/antiviral effects (notably colonic), enhanced antioxidant capacity, and improved intestinal morphology. Collectively, NG protects via augmented antioxidant activity, targeted anti-inflammatory/antiviral actions, and restored metabolic/structural integrity. NG possesses diverse sources and high safety, making it suitable for development as a green feed additive to repair intestinal damage following PEDV infection. Citrus processing byproducts are rich in NG; efficient extraction and purification enable waste resource utilization, reducing feed costs and aligning with sustainable farming trends.

## Figures and Tables

**Table 1:** Primer sequences for related genes.

Gene Name	Forward (5′-3′)	Reverse (5′-3′)	Accession Number
*ACAA1*	GGGAGAAGCAGGATACCTTTG	CATTGCCCTTGTCATCGTAGA	XM_021071664
*ACADL*	GGATGGAAGTGACTGGATTCTC	GAGAGCGAGCTTCACGATTT	NM_213897
*ACSL3*	TTTTGCTGTCCCGTTGGTC	GTATCCACCTTCTTCCCAGTTCTTT	NM_001143698
*APOA1*	CCTTGGCTGTGCTCTTCCTC	ACGGTGGCAAAATCCTTCAC	NM_214398
*APOA4*	ACCCAGCAGCTCAACACTCTC	GAGTCCTTGGTCAGGCGTTC	NM_214388
*APOB*	GGGATGATGGCACAGGTTACA	TGACGTGGACTTGGTGCTTT	NM_001375388
*APOC3*	CTAACCAGCGTGAAGGAGTC	CAGAAGTCGGTGAACTTGCC	NM_001002801
*AQP10*	GGGCGTTATACTAGCCATCTAC	CCAACTGCACCAAGGAGTAA	NM_001128454
*AQP7*	GAGTTCTTGGCCGAGTTCAT	CCCAACACGTACACAGGAAA	NM_001113438
*AREG*	GAGTACGATAACGAACCGCACA	TTTCCACTTTTGCCTCCCTTT	NM_214376
*ASAH2*	ATAGAGCACCTACAGGCAAAC	TCGGGTTAGCACCTACAAATAC	XM_005671250
*CLCA4*	ACAGCGTTTGAGGTGGTTAG	ATGATGGCCCCACTTTGTTT	XM_001926978
*CXCL2*	CGGAAGTCATAGCCACTCTCAA	CAGTAGCCAGTAAGTTTCCTCCATC	NM_001001861
*CYP2J34*	TGAGGCTGTTGGATGAAGTC	TGAAGAGGGTTTGGTGGG	NM_001244633
*CYP3A22*	AGCTCCTAAGATTTGATTTCCTCG	CCACTCGGCGCATTTGTT	NM_001195509
*DGAT1*	GCTGGCTCTGATGGTCTACG	GTAGAGATCTGCAGAAGCGGC	XM_005655311
*ENPP7*	CTGCCTTATCACACCACACT	CGCCTTGGTAGGTGACATT	XM_013980746
*FABP2*	GAAACTTGCAGCTCATGACAAT	GTCTGCGAGGCTGTAGTTAAA	NM_001031780
*FASN*	ACACCTTCGTGCTGGCCTAC	ATGTCGGTGAACTGCTGCAC	NM_001099930
*GPX2*	CCGGGACTTCACCCAACTC	CGGACGTACTTGAGGCTGTT	NM_001115136
*GSTO2*	GCCTTGAGATGTGGGAGAGAA	AAGATGGTGTTCTGATAGCCAAGA	XM_001927288
*HSD17B6*	TCAGATGTCCTGAGATGTGAGC	TCCAGACTTGCTTCATTGCCT	XM_005663895
*IFN-β*	AGCAGATCTTCGGCATTCTC	GTCATCCATCTGCCCATCAA	NM_001003923
*IL-1β*	CAACGTGCAGTCTATGGAGT	GAGGTGCTGATGTACCAGTTG	NM_214055
*IL-8*	TTCGATGCCAGTGCATAAATA	CTGTACAACCTTCTGCACCCA	NM_213867
*ISG15*	AGCATGGTCCTGTTGATGGTG	CAGAAATGGTCAGCTTGCACG	NM_001128469
*KCNJ13*	ATGGATGTGTCGCTGGTCTTT	CACAACTGCTTGCCTTTACGAG	XM_001926506
*MEP1A*	AAGCTGGTCAAGATGAAGACCT	TTTGAGTTCTGGGGATCACCTT	XM_001928416
*MME*	CACAACATCAGAAACAGCGACA	GGCAATCAAATCCTCAACCAC	XM_021069694
*MMP13*	AGTTTGGCCATTCCTTAGGTCTTG	GGCTTTTGCCAGTGTAGGTATAGAT	XM_003129808
*MMP7*	GGTGGCAGCATAGGCATTAAC	TCCGTAGGTTGGATACATCACAG	NM_001348795
*MTTP*	CCGTCGAGTTCTGAAGGAAAT	GAATGCCAGAACCAGAGTAGAG	NM_214185
*MUC5AC*	GTCAATGGCCGCACAATTCAG	CATCGTGGGAGAGGAACTCG	XM_021082583
*MX1*	AGTGCGGCTGTTTACCAAG	TTCACAAACCCTGGCAACTC	NM_214061
*NHE3*	AAGTACGTGAAGGCCAACATCTC	TTCTCCTTGACCTTGTTCTCGTC	XM_021077062
*OASL*	GGCACCCCTGTTTTCCTCT	AGCACCGCTTTTGGATGG	NM_001031790
*PCK1*	CGGGATTTCGTGGAGA	CCTCTTGATGACACCCTCT	NM_001123158
*PEDV M*	TCCCGTTGATGAGGTGAT	AGGATGCTGAAAGCGAAAA	KT021228
*PEDV N*	TTGGTGGTAATGTGGCTGTTC	TGGTTTCACGCTTGTTCTTCTT	KT021228
*PEDV S*	CTCTCTGGTACAGGCAGCAC	GCTCACGTAGAGTCAAGGCA	KT021228
*PLA2G3*	ACTCTGCTGGGAACTCATCT	GGTAGTTTCGGATGCCATAGTT	XM_021072944
*REG3G*	CTGTCTCAGGTCCAAGGTGAAG	CAAGGCATAGCAGTAGGAAGCA	XM_005662419
*RPL19*	AACTCCCGTCAGCAGATCC	AGTACCCTTCCGCTTACCG	XM_003131509
*RSAD2*	CCCCACTAGCGTCAATTACC	TGATCTTCTCCATACCCGCT	NM_213817
*S100A8*	AACTCTGTTTCGGGGAGACC	CGCGTAGATGGCGTGGTAA	NM_001160271
*S100A9*	CCAGGATGTGGTTTATGGCTTTC	CGGACCAAATGTCGCAGA	XM_013997035
*SCARB1*	CTTCGTGAACCGCACTGTTG	CCCGGAATCGGAGTTGTTGA	NM_213967
*SI*	ATGTCCGTGGTGGTCATATTC	TTTCCTTGTGCCGTCTGATTA	XM_021069750
*SLC27A4*	TGGAAAGGCGAGAACGTGT	CAGCAGAGTGGACAGTGAGCA	XM_021069609
*SLC5A1*	GTCATCTACTTCGTGGTGGTG	ACCCAAATCAGAGCATTCCATT	NM_001164021
*TRPM6*	TACGGGAAGAGATGTGGTGT	CGCCTGAGCTTCATCTCATT	XM_021064975
*TRPV6*	AGGAGCTGGTGAGCCTCAAGT	GGGGTCAGTTTGGTTGTTGG	NM_001436069

*ACAA1*, Acetyl-CoA Acyltransferase 1; *ACADL*, Acyl-CoA Dehydrogenase, Long Chain; *ACSL3*, Acyl-CoA Synthetase Long Chain Family Member 3; *APOA1*, Apolipoprotein A1; *APOA4*, Apolipoprotein A4; *APOB*, Apolipoprotein B; *APOC3*, Apolipoprotein C3; *AQP10*, Aquaporin 10; *AQP7*, Aquaporin 7; *AREG*, Amphiregulin; *CLCA4*, Chloride Channel Accessory 4; *ASAH2*, N-Acylsphingosine Amidohydrolase 2; *CXCL2*, C-X-C Motif Chemokine Ligand 2; *CYP2J34*, Cytochrome P450 Family 2 Subfamily J Member 34; *CYP3A22*, Cytochrome P450 Family 3 Subfamily A Member 22; *DGAT1*, Diacylglycerol O-Acyltransferase 1; *ENPP7*, Ectonucleotide Pyrophosphatase/Phosphodiesterase Family Member 7; *FABP2*, Fatty Acid Binding Protein 2, Intestinal; *FASN*, Fatty Acid Synthase; *GPX2*, Glutathione Peroxidase 2; *GSTO2*, Glutathione S-Transferase Omega 2; *HSD17B6*, Hydroxysteroid 17-Beta Dehydrogenase 6; *IFN-β*, Interferon Beta; *IL-1β*, Interleukin 1 Beta; *IL-8*, Interleukin 8; *ISG15*, Interferon Stimulated Gene 15; *KCNJ13*, Potassium Inwardly Rectifying Channel Subfamily J Member 13; *MEP1A*, Meprin A Subunit Alpha; *MME*, Membrane Metalloendopeptidase; *MMP13*, Matrix Metallopeptidase 13; *MMP7*, Matrix Metallopeptidase 7; *MTTP*, Microsomal Triglyceride Transfer Protein; *MUC5AC*, Mucin 5AC, Oligomeric Mucus/Gel-Forming; *MX1*, MX Dynamin Like GTPase 1; *NHE3*, Sodium Hydrogen Exchanger 3; *OASL*, 2′,5′-Oligoadenylate Synthetase Like; *PCK1*, Phosphoenolpyruvate Carboxykinase 1; *PEDV M*, Porcine Epidemic Diarrhea Virus Membrane Protein; *PEDV N*, Porcine Epidemic Diarrhea Virus Nucleocapsid Protein; *PEDV S*, Porcine Epidemic Diarrhea Virus Spike Protein; *PLA2G3*, Phospholipase A2 Group III; *REG3G*, Regenerating Family Member 3 Gamma; *RPL19*, Ribosomal Protein L19; *RSAD2*, Radical S-Adenosyl Methionine Domain Containing 2; *S100A8*, S100 Calcium Binding Protein A8; *S100A9*, S100 Calcium Binding Protein A9; *SCARB1*, Scavenger Receptor Class B Member 1; *SI*, Sucrase-Isomaltase; *SLC27A4*, Solute Carrier Family 27 Member 4; *SLC5A1*, Solute Carrier Family 5 Member 1; *TRPM6*, Transient Receptor Potential Cation Channel Subfamily M Member 6; *TRPV6*, Transient Receptor Potential Cation Channel Subfamily V Member 6.

**Table 2:** Piglets’ daily feed intake.

	Group	Daily Feed Intake
D4	CON	3600 g/day
	PEDV	3600 g/day
	NG + PEDV	3600 g/day
D5	CON	3600 g/day
	PEDV	3600 g/day
	NG + PEDV	3600 g/day
D6	CON	3600 g/day
	PEDV	3600 g/day
	NG + PEDV	3600 g/day
D7	CON	3600 g/day
	PEDV	3600 g/day
	NG + PEDV	3600 g/day
D8	CON	3600 g/day
	PEDV	3600 g/day
	NG + PEDV	3600 g/day
D9	CON	3600 g/day
	PEDV	3600 g/day
	NG + PEDV	3600 g/day
D10	CON	3600 g/day
	PEDV	3600 g/day
	NG + PEDV	3600 g/day

Daily feed intake data was calculated using the following formula: Group daily feed intake (g/day) = Group feed administered − Group feed leftovers − Group feed discarded. D4, Day 4 of the experiment. D5, Day 5 of the experiment. D6, Day 6 of the experiment. D7, Day 7 of the experiment. D8, Day 8 of the experiment. D9, Day 9 of the experiment. D10, Day 10 of the experiment. CON, the control group. PEDV, the PEDV infection group. NG + PEDV, the NG + PEDV infection group (*n* = 6).

**Table 3:** Effect of NG on ADG, diarrhea score, and diarrhea rate in PEDV-infected piglets.

Item	CON	PEDV	NG + PEDV	*p*-Value
ADG (g) (4–8)	86.04 ± 10.28	87.08 ± 16.20	89.54 ± 18.08	0.161
ADG (g) (8–11)	141.67 ± 11.20	51.28 ± 37.73	80.58 ± 23.36	0.079

ADG, Average daily weight gain. ADG was calculated as the difference between the final body weight and initial body weight divided by the number of feeding days during each experimental phase (4–8 days of age and 8–11 days of age). CON, the control group. PEDV, the PEDV infection group. NG + PEDV, the NG + PEDV infection group. Data are presented as means ± standard error of the mean for each group (*n* = 6). A *p*-value of <0.05 was deemed statistically significant.

**Table 4:** Effect of NG on serum DAO and D-xylose levels in PEDV-infected piglets.

Item	CON	PEDV	NG + PEDV	*p*-Value
DAO (U/L)	7.55 ± 0.87	8.36 ± 0.37	7.57 ± 0.91	0.694
D-xylose (mmol/L)	0.99 ± 0.04 ^a^	0.56 ± 0.02 ^b^	0.61 ± 0.08 ^b^	<0.001

DAO, Diamine oxidase. CON, the control group. PEDV, the PEDV infection group. NG + PEDV, the NG + PEDV infection group. Data are presented as means ± standard error of the mean for each group (*n* = 6). ^a, b^ Values within a column not sharing a common superscript letter indicate significant difference at *p* < 0.05.

**Table 5:** Effect of NG on plasma biochemical parameters in PEDV-infected piglets.

Item	CON	PEDV	NG + PEDV	*p*-Value
TB (μmol/L)	1.89 ± 0.3 ^a^	1.01 ± 0.16 ^b^	2.29 ± 0.29 ^a^	0.01
TP (g/L)	53.71 ± 1.21 ^b^	63.61 ± 1.75 ^a^	66.52 ± 2.36 ^a^	<0.001
ALB (g/L)	28.58 ± 0.75 ^b^	30.88 ± 1.45 ^b^	35.12 ± 0.89 ^a^	0.002
AST (U/L)	33.67 ± 4.28	30.83 ± 4.1	33.17 ± 3.35	0.864
ALT (U/L)	31.33 ± 3.39 ^b^	39.33 ± 2.11 ^ab^	46.67 ± 4.62 ^a^	0.025
ALP (U/L)	826 ± 79.63 ^a^	553.17 ± 49.05 ^b^	711.67 ± 62.05 ^ab^	0.03
TC (mmol/L)	3.84 ± 0.4	3.31 ± 0.16	4.43 ± 0.62	0.224
TG (mmol/L)	0.53 ± 0.05 ^b^	0.63 ± 0.05 ^b^	0.91 ± 0.09 ^a^	0.003
GLU (mmol/L)	6.85 ± 0.38	6.45 ± 0.42	7.32 ± 0.49	0.388
Ca (g/L)	4.93 ± 0.29	4.29 ± 0.11	4.73 ± 0.13	0.084
P (mg/dL)	2.66 ± 0.14	2.66 ± 0.1	2.69 ± 0.22	0.993
CREA (μmol/L)	64.66 ± 3.67 ^b^	75.83 ± 2.92 ^ab^	87.23 ± 3.61 ^a^	0.001
HDL (mmol/L)	1.77 ± 0.22	1.28 ± 0.09	1.67 ± 0.21	0.172
LDL (mmol/L)	1.22 ± 0.11 ^b^	1.23 ± 0.09 ^b^	1.81 ± 0.21 ^a^	0.016
BUN (mmol/L)	1.93 ± 0.22 ^b^	6.58 ± 0.57 ^a^	8.12 ± 0.69 ^a^	<0.001
GGT (U/L)	45 ± 5.54	48 ± 2.92	58.33 ± 6.58	0.203
CK (U/L)	413.67 ± 46.2 ^a^	210.5 ± 17.74 ^b^	104.67 ± 13.79 ^b^	<0.001
LDH (U/L)	772.52 ± 25.13	661.37 ± 24.04	727.58 ± 55.77	0.149

TB, Total Bilirubin; TP, Total Protein; ALB, Albumin; AST, Aspartate Aminotransferase; ALT, Alanine Aminotransferase; ALP, Alkaline Phosphatase; TC, Total Cholesterol; TG, Triglycerides; GLU, Glucose; Ca, Calcium; P, Phosphorus; CREA, Creatinine; HDL, High-Density Lipoprotein; LDL, Low-Density Lipoprotein; BUN, Blood Urea Nitrogen; GGT, Gamma-Glutamyl Transferase; CK, Creatine Kinase; LDH, Lactate Dehydrogenase. CON, the control group. PEDV, the PEDV infection group. NG + PEDV, the NG + PEDV infection group. Data are presented as means ± standard error of the mean for each group (*n* = 6). ^a, b^ Values within a column not sharing a common superscript letter indicate significant difference at *p* < 0.05.

**Table 6:** Effect of NG on hematological parameters of PEDV-infected piglets.

Item	CON	PEDV	NG + PEDV	*p*-Value
WBC (10^9^/L)	9.37 ± 0.96	8.91 ± 0.45	10.72 ± 1.11	0.799
Neu (10^9^/L)	2.91 ± 0.3	2.77 ± 0.28	2.84 ± 0.34	0.411
Lym (10^9^/L)	6.44 ± 0.53	5.26 ± 0.4	5.71 ± 0.51	0.830
Mon (10^9^/L)	0.35 ± 0.04 ^b^	0.72 ± 0.05 ^a^	0.83 ± 0.1 ^a^	0.008
Eos (10^9^/L)	0.08 ± 0.01	0.09 ± 0.01	0.05 ± 0.01	0.142
Neu% (%)	31.9 ± 2.85	30.93 ± 2.38	38.05 ± 4.68	0.414
Lym% (%)	60.23 ± 4.57	59.08 ± 3.11	52.28 ± 4.71	0.374
Mon% (%)	3.88 ± 0.43 ^b^	8.97 ± 1.06 ^a^	9.07 ± 1.14 ^a^	0.008
Eos% (%)	0.68 ± 0.07	0.82 ± 0.04	0.6 ± 0.08	0.173
RBC (10^12^/L)	5.23 ± 0.38	6.31 ± 0.22	6.18 ± 0.75	0.275
HGB (g/L)	108.33 ± 7.85	131.67 ± 2.44	123 ± 13.78	0.231
HCT (%)	33.62 ± 2.35	41.17 ± 1.16	37.58 ± 4.24	0.212
MCV (fL)	64.47 ± 1.13	65.35 ± 1.64	61.45 ± 1.99	0.238
MCH (pg)	20.75 ± 0.47	20.92 ± 0.61	20.1 ± 0.53	0.541
MCHC (g/L)	322.33 ± 3.72	320.17 ± 4.54	327.33 ± 2.26	0.383
RDW-CV (%)	23.8 ± 0.56	23.38 ± 0.68	23.98 ± 0.69	0.799
RDW-SD (fL)	55.78 ± 1.33	55.73 ± 2.28	53.8 ± 2.76	0.772
PLT (10^9^/L)	453.5 ± 51.04	451.14 ± 56.68	334.22 ± 39.44	0.371
MPV (fL)	9.9 ± 0.28	9.83 ± 0.43	9.95 ± 0.33	0.973
PDW (%)	15.4 ± 0.11	15.58 ± 0.1	15.28 ± 0.09	0.151
PCT (%)	0.45 ± 0.05	0.39 ± 0.03	0.33 ± 0.04	0.412

WBC, White Blood Cells; Neu, Neutrophils; Lym, Lymphocytes; Mon, Monocytes; Eos, Eosinophils; RBC, Red Blood Cells; HGB, Hemoglobin; HCT, Hematocrit; MCV, Mean Corpuscular Volume; MCH, Mean Corpuscular Hemoglobin; MCHC, Mean Corpuscular Hemoglobin Concentration; RDW-CV, Red Cell Distribution Width—Coefficient of Variation; RDW-SD, Red Cell Distribution Width—Standard Deviation; PLT, Platelets; MPV, Mean Platelet Volume; PDW, Platelet Distribution Width; PCT, Plateletcrit. CON, the control group. PEDV, the PEDV infection group. NG + PEDV, the NG + PEDV infection group. Data are presented as means ± standard error of the mean for each group (*n* = 6). ^a, b^ Values within a column not sharing a common superscript letter indicate significant difference at *p* < 0.05.

## Data Availability

All data presented in this research is included in the article. Further inquiries can be directed to the corresponding author.

## Abbreviations

ACAA1	Acetyl-CoA Acyltransferase 1
ACADL	Acyl-CoA Dehydrogenase, Long Chain
ACSL3	Acyl-CoA Synthetase Long Chain Family Member 3
APOA1	Apolipoprotein A1
APOA4	Apolipoprotein A4
APOB	Apolipoprotein B
APOC3	Apolipoprotein C3
AQP10	Aquaporin 10
AQP7	Aquaporin 7
AREG	Amphiregulin
ASAH2	N-Acylsphingosine Amidohydrolase 2
CLCA4	Chloride Channel Accessory 4
CXCL2	C-X-C Motif Chemokine Ligand 2
CYP2J34	Cytochrome P450 Family 2 Subfamily J Member 34
CYP3A22	Cytochrome P450 Family 3 Subfamily A Member 22
DGAT1	Diacylglycerol O-Acyltransferase 1
ENPP7	Ectonucleotide Pyrophosphatase/Phosphodiesterase Family Member 7
FABP2	Fatty Acid Binding Protein 2, Intestinal
FASN	Fatty Acid Synthase
GPX2	Glutathione Peroxidase 2
GSTO2	Glutathione S-Transferase Omega 2
HSD17B6	Hydroxysteroid 17-Beta Dehydrogenase 6
IFN-β	Interferon Beta
IL-1β	Interleukin 1 Beta
IL-8	Interleukin 8
ISG15	Interferon Stimulated Gene 15
KCNJ13	Potassium Inwardly Rectifying Channel Subfamily J Member 13
MEP1A	Meprin A Subunit Alpha
MME	Membrane Metalloendopeptidase
MMP13	Matrix Metallopeptidase 13
MMP7	Matrix Metallopeptidase 7
MTTP	Microsomal Triglyceride Transfer Protein
MUC5AC	Mucin 5AC, Oligomeric Mucus/Gel-Forming
MX1	MX Dynamin Like GTPase 1
NHE3	Sodium Hydrogen Exchanger 3
OASL	2′,5′-Oligoadenylate Synthetase Like
PCK1	Phosphoenolpyruvate Carboxykinase 1
PEDV M	Porcine Epidemic Diarrhea Virus Membrane Protein
PEDV N	Porcine Epidemic Diarrhea Virus Nucleocapsid Protein
PEDV S	Porcine Epidemic Diarrhea Virus Spike Protein
PLA2G3	Phospholipase A2 Group III
REG3G	Regenerating Family Member 3 Gamma
RPL19	Ribosomal Protein L19
RSAD2	Radical S-Adenosyl Methionine Domain Containing 2
S100A8	S100 Calcium Binding Protein A8
S100A9	S100 Calcium Binding Protein A9
SCARB1	Scavenger Receptor Class B Member 1
SI	Sucrase-Isomaltase
SLC27A4	Solute Carrier Family 27 Member 4
SLC5A1	Solute Carrier Family 5 Member 1
TRPM6	Transient Receptor Potential Cation Channel Subfamily M Member 6
TRPV6	Transient Receptor Potential Cation Channel Subfamily V Member 6

## References

[1] Tang X., Xiong K., Fang R., Li M. (2022). Weaning stress and intestinal health of piglets: A review. Front. Immunol..

[2] Liu Q., Wang H.Y. (2021). Porcine enteric coronaviruses: An updated overview of the pathogenesis, prevalence, and diagnosis. Vet. Res. Commun..

[3] Du J., Luo J., Yu J., Mao X., Luo Y., Zheng P., He J., Yu B., Chen D. (2019). Manipulation of Intestinal Antiviral Innate Immunity and Immune Evasion Strategies of Porcine Epidemic Diarrhea Virus. BioMed Res. Int..

[4] Lei J., Miao Y., Bi W., Xiang C., Li W., Zhang R., Li Q., Yang Z. (2024). Porcine Epidemic Diarrhea Virus: Etiology, Epidemiology, Antigenicity, and Control Strategies in China. Animals.

[5] Li Z., Ma Z., Li Y., Gao S., Xiao S. (2020). Porcine epidemic diarrhea virus: Molecular mechanisms of attenuation and vaccines. Microb. Pathog..

[6] Yang C., Chowdhury M.A., Huo Y., Gong J. (2015). Phytogenic compounds as alternatives to in-feed antibiotics: Potentials and challenges in application. Pathogens.

[7] Biswas S., Ahn J.M., Kim I.H. (2024). Assessing the potential of phytogenic feed additives: A comprehensive review on their effectiveness as a potent dietary enhancement for nonruminant in swine and poultry. J. Anim. Physiol. Anim. Nutr..

[8] Gorinstein S., Leontowicz H., Leontowicz M., Krzeminski R., Gralak M., Delgado-Licon E., Martinez Ayala A.L., Katrich E., Trakhtenberg S. (2005). Changes in plasma lipid and antioxidant activity in rats as a result of naringin and red grapefruit supplementation. J. Agric. Food Chem..

[9] Ekinci Akdemir F.N., Gülçin İ., Karagöz B., Soslu R., Alwasel S.H. (2016). A comparative study on the antioxidant effects of hesperidin and ellagic acid against skeletal muscle ischemia/reperfusion injury. J. Enzym. Inhib. Med. Chem..

[10] Chen R., Qi Q.L., Wang M.T., Li Q.Y. (2016). Therapeutic potential of naringin: An overview. Pharm. Biol..

[11] Chen N., Gao H.X., He Q., Zeng W.C. (2023). Insight into property, function, and digestion of potato starch modified by phenolic compounds with varying structures. J. Food Sci..

[12] Babacanoglu Z., Acar G., Aladag T., Baltaci S.B., Mogulkoc R., Baltaci A.K. (2025). Naringin Supplementation Reduces Inflammatory Processes in the Cerebellum in Brain Ischemia of Rats. Curr. Top. Med. Chem..

[13] Bayram P., Aksak Karamese S., Ozdemir B., Salum C., Erol H.S., Karamese M. (2023). Two flavonoids, baicalein and naringin, are effective as anti-inflammatory and anti-oxidant agents in a rat model of polymicrobial sepsis. Immunopharmacol. Immunotoxicol..

[14] Vincer B., Sindya J., Rajanathadurai J., Perumal E. (2024). Exploring the Cytotoxic and Anticancer Potential of Naringin on Oral Cancer Cell Line. Cureus.

[15] Poudineh M., Ghotbi T., Azizi F., Karami N., Zolfaghari Z., Gheisari F., Hormozi M., Poudineh S. (2022). Neuropharmaceutical Properties of Naringin Against Alzheimer’s and Parkinson’s Diseases: Naringin Protection Against AD and PD. Galen Med. J..

[16] Jing X.H., Zhao G.Y., Wang G.B., Huang Q.L., Zou W.S., Huang L.N., Li W., Qiu Z.Y., Xin R.H. (2024). Naringin alleviates pneumonia caused by Klebsiella pneumoniae infection by suppressing NLRP3 inflammasome. Biomed. Pharmacother..

[17] Cao R., Wu X., Guo H., Pan X., Huang R., Wang G., Liu J. (2021). Naringin Exhibited Therapeutic Effects against DSS-Induced Mice Ulcerative Colitis in Intestinal Barrier-Dependent Manner. Molecules.

[18] Stabrauskiene J., Kopustinskiene D.M., Lazauskas R., Bernatoniene J. (2022). Naringin and Naringenin: Their Mechanisms of Action and the Potential Anticancer Activities. Biomedicines.

[19] Gong M., Xia X., Chen D., Ren Y., Liu Y., Xiang H., Li X., Zhi Y., Mo Y. (2023). Antiviral activity of chrysin and naringenin against porcine epidemic diarrhea virus infection. Front. Vet. Sci..

[20] Jain A.S., Sushma P., Dharmashekar C., Beelagi M.S., Prasad S.K., Shivamallu C., Prasad A., Syed A., Marraiki N., Prasad K.S. (2021). In silico evaluation of flavonoids as effective antiviral agents on the spike glycoprotein of SARS-CoV-2. Saudi J. Biol. Sci..

[21] Aati H.Y., Ismail A., Rateb M.E., AboulMagd A.M., Hassan H.M., Hetta M.H. (2022). Garcinia cambogia Phenolics as Potent Anti-COVID-19 Agents: Phytochemical Profiling, Biological Activities, and Molecular Docking. Plants.

[22] Ali A.M., Kunugi H. (2021). Propolis, Bee Honey, and Their Components Protect against Coronavirus Disease 2019 (COVID-19): A Review of In Silico, In Vitro, and Clinical Studies. Molecules.

[23] Xu Z., Zhang Q., Wu M., Zhang Y., Li Z., Li H., Yu C., Zhang X., Zhao D., Wang L. (2024). Lactobacillus rhamnosus GG powder supplementation alleviates intestinal injury in piglets challenged by porcine epidemic diarrhea virus. Front. Cell. Infect. Microbiol..

[24] Wang Q., Wang J., Qi R.L., Qiu X.Y., Sun Q., Huang J.X. (2020). Naringin supplementation affects performance, carcass traits, meat quality and oxidative stability of finishing pigs. S. Afr. J. Anim. Sci..

[25] Goodarzi Boroojeni F., Männer K., Zentek J. (2018). The impacts of Macleaya cordata extract and naringin inclusion in post-weaning piglet diets on performance, nutrient digestibility and intestinal histomorphology. Arch. Anim. Nutr..

[26] Lange C.D. (2014). Nutrient Requirements of Swine (2012).

[27] Alam F., Badruddeen B., Kumar Kharya A., Juber A., Irfan Khan M. (2022). Relationship between the Dose administered and Toxicity level after Acute Oral Exposure to Lupeol and Naringin combination in rats. Res. J. Pharm. Technol..

[28] Li P., Wu H., Wang Y., Peng W., Su W. (2020). Toxicological evaluation of naringin: Acute, subchronic, and chronic toxicity in Beagle dogs. Regul. Toxicol. Pharmacol..

[29] Rebello C.J., Beyl R.A., Lertora J.J.L., Greenway F.L., Ravussin E., Ribnicky D.M., Poulev A., Kennedy B.J., Castro H.F., Campagna S.R. (2020). Safety and pharmacokinetics of naringenin: A randomized, controlled, single-ascending-dose clinical trial. Diabetes Obes. Metab..

[30] Kwon Y.K., Son K.H., Choi D., Bok S.H., Kim S.U., Moon S.S., Bae K.H., Hwang I., Ahn J.A., Lee E.S. (1998). Naringin and Naringenin as 3-Hydroxy-3-Methylglutaryl CoA(HMG-CoA) Reductase Inhibitor. Canadian Patent.

[31] Frankel W.L., Zhang W., Afonso J., Klurfeld D.M., Don S.H., Laitin E., Deaton D., Furth E.E., Pietra G.G., Naji A. (1993). Glutamine enhancement of structure and function in transplanted small intestine in the rat. JPEN J. Parenter. Enter. Nutr..

[32] Fu W.J., Stromberg A.J., Viele K., Carroll R.J., Wu G. (2010). Statistics and bioinformatics in nutritional sciences: Analysis of complex data in the era of systems biology. J. Nutr. Biochem..

[33] Hou Y., Wang L., Zhang W., Yang Z., Ding B., Zhu H., Liu Y., Qiu Y., Yin Y., Wu G. (2012). Protective effects of N-acetylcysteine on intestinal functions of piglets challenged with lipopolysaccharide. Amino Acids.

[34] Zhang Q., Wang S., Wu M., Tan Z., Wu T., Yi D., Wang L., Zhao D., Hou Y. (2025). Multi-omics profiling reveals Poria cocos polysaccharides mitigate PEDV-induced intestinal injury by modulating lipid metabolism in piglets. J. Anim. Sci. Biotechnol..

[35] Tannous S., Naim H.Y. (2024). Impaired digestive function of sucrase-isomaltase in a complex with the Greenlandic sucrase-isomaltase variant. Biochim. Biophys. Acta Mol. Basis Dis..

[36] Alfalah M., Keiser M., Leeb T., Zimmer K.P., Naim H.Y. (2009). Compound heterozygous mutations affect protein folding and function in patients with congenital sucrase-isomaltase deficiency. Gastroenterology.

[37] Gonzalez-Rellan M.J., Fernández U., Parracho T., Novoa E., Fondevila M.F., da Silva Lima N., Ramos L., Rodríguez A., Serrano-Maciá M., Perez-Mejias G. (2023). Neddylation of phosphoenolpyruvate carboxykinase 1 controls glucose metabolism. Cell Metab..

[38] Zhu S., Ran J., Yang B., Mei Z. (2017). Aquaporins in Digestive System. Adv. Exp. Med. Biol..

[39] Kan Z., Zhang S., Liao G., Niu Z., Liu X., Sun Z., Hu X., Zhang Y., Xu S., Zhang J. (2023). Mechanism of *Lactiplantibacillus plantarum* regulating Ca(^2+^) affecting the replication of PEDV in small intestinal epithelial cells. Front. Microbiol..

[40] van der Wijst J., Blanchard M.G., Woodroof H.I., Macartney T.J., Gourlay R., Hoenderop J.G., Bindels R.J., Alessi D.R. (2014). Kinase and channel activity of TRPM6 are co-ordinated by a dimerization motif and pocket interaction. Biochem. J..

[41] Zhang J., Zhao D., Yi D., Wu M., Chen H., Wu T., Zhou J., Li P., Hou Y., Wu G. (2019). Microarray analysis reveals the inhibition of intestinal expression of nutrient transporters in piglets infected with porcine epidemic diarrhea virus. Sci. Rep..

[42] Xia P., Ji X., Yan L., Lian S., Chen Z., Luo Y. (2024). Roles of S100A8, S100A9 and S100A12 in infection, inflammation and immunity. Immunology.

[43] Wang Y., Che M., Xin J., Zheng Z., Li J., Zhang S. (2020). The role of IL-1β and TNF-α in intervertebral disc degeneration. Biomed. Pharmacother..

[44] Dissanayake W.M.N., Chandanee M.R., Lee S.M., Heo J.M., Yi Y.J. (2023). Change in intestinal alkaline phosphatase activity is a hallmark of antibiotic-induced intestinal dysbiosis. Anim. Biosci..

[45] Fan B., Zhou J., Zhao Y., Zhu X., Zhu M., Peng Q., Li J., Chang X., Shi D., Yin J. (2023). Identification of Cell Types and Transcriptome Landscapes of Porcine Epidemic Diarrhea Virus-Infected Porcine Small Intestine Using Single-Cell RNA Sequencing. J. Immunol..

[46] Hartkamp L.M., van Es I.E., Malvar Fernandez B., Tak P.P., Reedquist K.A. (2014). A1.51 The AGC kinases protein kinase B (PKB) and serum and glucocorticoid kinase (SGK) differentially regulate the metabolic activity and inflammatory activation of rheumatoid arthritis fibroblast-like synoviocytes. Ann. Rheum. Dis..

[47] Cordiano R., Di Gioacchino M., Mangifesta R., Panzera C., Gangemi S., Minciullo P.L. (2023). Malondialdehyde as a Potential Oxidative Stress Marker for Allergy-Oriented Diseases: An Update. Molecules.

[48] Li R., Shen M., Hu J., Liu J., Tian X., Li M., Li Z., Yi D., Wu T., Wang L. (2024). A combination of puerarin and poria cococs polysaccharide alleviates the excessive autophagy-caused jejunal injury by increasing serine dehydratase like (SDSL) levels in PEDV-infected piglets. J. Funct. Foods.

[49] Yen M.C., Chou S.K., Kan J.Y., Kuo P.L., Hou M.F., Hsu Y.L. (2018). Solute Carrier Family 27 Member 4 (SLC27A4) Enhances Cell Growth, Migration, and Invasion in Breast Cancer Cells. Int. J. Mol. Sci..

[50] Anaganti N., Valmiki S., Recacha R., Islam S., Farber S., Ruddock L., Hussain M.M. (2024). Bulky hydrophobic side chains in the β1-sandwich of microsomal triglyceride transfer protein are critical for the transfer of both triglycerides and phospholipids. J. Biol. Chem..

[51] Kohan A.B., Wang F., Lo C.M., Liu M., Tso P. (2015). ApoA-IV: Current and emerging roles in intestinal lipid metabolism, glucose homeostasis, and satiety. Am. J. Physiol. Gastrointest. Liver Physiol..

[52] Visser J., van Zwol W., Kuivenhoven J.A. (2022). Managing of Dyslipidaemia Characterized by Accumulation of Triglyceride-Rich Lipoproteins. Curr. Atheroscler. Rep..

[53] Edin M.L., Lih F.B., Hammock B.D., Thomson S., Zeldin D.C., Bishop-Bailey D. (2020). Vascular Lipidomic Profiling of Potential Endogenous Fatty Acid PPAR Ligands Reveals the Coronary Artery as Major Producer of CYP450-Derived Epoxy Fatty Acids. Cells.

[54] Lv K., Song J., Wang J., Zhao W., Yang F., Feiya J., Bai L., Guan W., Liu J., Ho C.T. (2024). Pterostilbene Alleviates Dextran Sodium Sulfate (DSS)-Induced Intestinal Barrier Dysfunction Involving Suppression of a S100A8-TLR-4-NF-κB Signaling Cascade. J. Agric. Food Chem..

[55] Hayase E., Hashimoto D., Nakamura K., Noizat C., Ogasawara R., Takahashi S., Ohigashi H., Yokoi Y., Sugimoto R., Matsuoka S. (2017). R-Spondin1 expands Paneth cells and prevents dysbiosis induced by graft-versus-host disease. J. Exp. Med..

[56] Chu F.F., Esworthy R.S. (1995). The expression of an intestinal form of glutathione peroxidase (GSHPx-GI) in rat intestinal epithelium. Arch. Biochem. Biophys..

[57] Chen J., Li F., Yang W., Jiang S., Li Y. (2021). Supplementation with Exogenous Catalase from Penicillium notatum in the Diet Ameliorates Lipopolysaccharide-Induced Intestinal Oxidative Damage through Affecting Intestinal Antioxidant Capacity and Microbiota in Weaned Pigs. Microbiol. Spectr..

